# BAR12: Bayesian Autoregressive Phase 1‐2 Design for Cell Therapy Trials With Manufacturing Changes

**DOI:** 10.1002/sim.70551

**Published:** 2026-04-20

**Authors:** Cheng‐Han Yang, Peter F. Thall, David Marin, Sheferaw Y. Belay, Ruitao Lin

**Affiliations:** ^1^ Department of Biostatistics The University of Texas MD Anderson Cancer Center Houston Texas USA; ^2^ Department of Stem Cell Transplantation and Cellular Therapy The University of Texas MD Anderson Cancer Center Houston Texas USA

## Abstract

An early phase dose‐finding trial of a new cell therapy may involve one or more manufacturing modifications made during the trial, known as “tweaks,” to improve the cell product quality. For example, a tweak may change the cell culture duration, cytokine cocktail, or donor criteria. Ideally, a tweak can be done without changing the treatment so much that the trial must be restarted as if the treatment were entirely new. However, a statistical design still should account for changes in the dose–response distribution due to the tweak. For such settings, we propose a Bayesian AutoRegressive Phase 1‐2 (BAR12) design that accounts for manufacturing tweaks during a phase 1‐2 trial by using a first order autoregressive model with spike‐and‐slab priors having components corresponding to pre‐ and post‐tweak distributions of toxicity and efficacy parameters. Simulations under a broad range of dose‐response functions were conducted to compare BAR12 to conventional phase 1‐2 designs that either assume the tweak had no effect and use all available data, or ignore the pre‐tweak data. The simulations show that BAR12 has superior operating characteristics, including higher probabilities of correct dose selection, allocation of more patients to optimal doses, and more efficient monitoring for identifying unsafe or ineffective doses.

## 1 Introduction

Early‐phase clinical trials of new cell therapies often present unique logistical and scientific challenges. The process of manufacturing a new cellular treatment requires identifying an appropriate cell source and generating a cell product that is sufficiently viable, potent, and safe to be used therapeutically. An important problem that may arise during a phase 1 or phase 1‐2 trial to identify an optimal dose of a new cell therapy is that one or more modifications of the manufacturing process, known as “tweaks”, may be made during the trial to obtain a higher‐quality cell product. Examples of tweaks include changes to the cell culture duration, changes in the cytokine cocktail used to grow the cells [1], addition of immunomodulators [2, 3], or changes of the criteria for cell donor selection [4, 5]. Ideally, a tweak does not make the treatment so different that a completely new clinical trial is required. It is more desirable if the ongoing study may integrate the improvements while still using the data from patients treated before a tweak. This practice introduces an important problem, however. In an early phase trial where cell doses are chosen adaptively for successive patient cohorts, if one or more tweaks are made during the trial then each patient's treatment is characterized by both the cell dose that they receive and the particular manufacturing process used to prepare their cellular therapy. This problem also may arise in trials of other types of novel engineered biotherapeutic agents, such as genes, antibodies, or proteins.

Requiring a trial to be terminated and a new protocol written and submitted for administrative and regulatory approval each time a biotherapy is tweaked would reduce the treatment development and evaluation process to an impractically slow pace. To address this issue, the U.S. Food and Drug Administration (FDA) issued guidance to support maintaining consistent clinical outcomes when one or more modifications to the manufacturing process are introduced during a trial [6]. If an early phase trial is not terminated each time a biotherapy product is tweaked, then a key question is how a statistical dose–response model and design may be formulated to account for one or more interim tweaks while using the data from all patients.

To address this problem, we propose a new Bayesian AutoRegressive Phase 1‐2 design, BAR12, that chooses doses for successive patient cohorts based on toxicity and response, while accounting for tweaks in the manufacturing process made during the trial. To characterize changes in dose effects between successive stages of the trial determined by tweaks, BAR12 relies on a Bayesian autoregressive model for the prior of the stage‐specific dose‐toxicity and dose–response parameters. A first‐order autoregressive (AR1) structure is assumed for the prior of stage‐specific parameters in which each conditional post‐ versus pre‐tweak distribution is a spike‐and‐slab mixture of two normal distributions, with respective means equal to the parameters of the previous stage and non‐informative baseline parameters. This modeling strategy may be applied to modify any parametric model‐based phase 1‐2 design to account for tweaks.

Many model‐based phase 1‐2 designs that choose doses for successive patient cohorts using both efficacy and toxicity outcomes have been proposed. These include a design that uses a trinary outcome to find a best dose satisfying both safety and efficacy requirements Thall and Russell [7], the Efficacy‐Toxicity probability trade‐off based design [8, 9], the simple toxicity and efficacy interval design [10], and the Bayesian Optimal interval design [11]. Reviews are given by Yuan et al. [12], Yan et al. [13], and Yuan et al. [14]. Our new design addresses a novel research question that has not been explored in the existing literature. Additionally, we propose an adaptive randomization strategy for patient allocation during the post‐tweak stages to facilitate a less biased dose comparison and selection, leveraging the ample safety data collected prior to the tweak.

To focus on the problem of accounting for the joint effects of cell doses and manufacturing changes on clinical outcomes, we will illustrate BAR12 using a simplified version of the generalized phase 1‐2 (Gen 1‐2) design proposed by Thall et al. [15]. The Gen 1‐2 design was motivated by a trial to optimize the dose of CD70 chimeric antigen receptor (CAR) natural killer (NK) cells given as immunotherapy for patients with recurrent or refractory cancer, studying the four doses 5.0 × $\left(10^{6},10^{7},10^{8},10^{9}\right)$ CD70 CAR NK cells. To account for the effects of tweaks, a new stage of the trial begins each time a tweak is made. We will assume that toxicity and response are binary variables observed within 30 days from start of therapy. While patient heterogeneity may be very important in phase 1‐2, [16, 17, 18], in order to focus on the problem at hand we also assume that patients are homogeneous with regard to effects of the manufacturing process and dose. For our illustration, we will construct a utility‐based phase 1‐2 design with an underlying model that includes stage‐specific dose–response distributions with a hierarchical mixture prior of the form described above. Trial conduct will use rules based on these distributions for monitoring dose acceptability and choosing doses for patients enrolled in each stage. Extensions to accommodate more complex clinical outcomes or patient heterogeneity, or to incorporate long term treatment success by conducting a Gen 1‐2 trial, will be discussed briefly in Section 6.

The remainder of the paper is organized as follows. A Bayesian autoregressive dose–response model with a spike‐and‐slab prior that accounts for tweaks in the manufacturing process during a trial is presented in Section 2. Section 3 describes adaptive dose acceptability criteria and posterior mean utilities, each accounting for tweaks, that are used to screen doses for safety and efficacy and choose a best dose during each stage of the trial. Results of a simulation study to evaluate the BAR12 design's operating characteristics (OCs) are presented in Section 4. Section 5 presents sensitivity analyses, and we close with a discussion in Section 6.

## 2 Probability Model

### 2.1 Bayesian Autoregressive Dose–Response Model

A BAR12 design may be constructed by modifying any phase 1‐2 dose–response model and design to account for differences in cell dose effects between stages of the trial determined by tweaks. We index trial stages by k=1,⋯,K. We define stage 1 of the trial as the period during which the initial manufacturing process is used to produce the cell therapy product. Stage 2 refers to the period following the implementation of a modified manufacturing process. If additional manufacturing tweaks are introduced, subsequent periods are referred to as stage 3, and so on. In most settings, a practical limitation is K≤3. Denote the stage‐specific model parameter vectors by $\theta_{1},\cdots ,\theta_{K}$. A key property of our assumed model is that the $\theta_{k}$'s are associated with each other, so estimation of $\theta_{k}$ may use the data from stages 1,⋯,k−1 as well as stage k to borrow strength. To implement this, we assume the following Bayesian autoregressive model. Let $\theta_{0}$ be a parameter vector with a non‐informative prior corresponding to little knowledge about cell dose effects before the trial is begun. For stage 1, before any tweaks, we assume that the prior $p\left(\theta_{1}|\theta_{0}\right)$ has mean $\theta_{0}$ and is non‐informative. At the start of each new stage k≥2, we assume a conditional prior on $\theta_{k}$ that follows the AR1 structure

$$p\left(\theta_{k}|\theta_{1},\cdots ,\theta_{k-1},\theta_{0}\right)=p\left(\theta_{k}|\theta_{k-1},\theta_{0}\right),\;\text{for}\;k=2,\cdots ,K.$$

To borrow information between successive stages, for each stage k≥2 we assume that $p\left(\theta_{k}|\theta_{k-1},\theta_{0}\right)$ is a spike‐and‐slab distribution that is a mixture of an informative spike component with mean $\theta_{k-1}$ and a non‐informative slab component with mean $\theta_{0}.$ This allows the posterior distribution of each $\theta_{k}$ to be evaluated using the data from stages k and k−1, rather than only stage k. In turn, this provides a practical, scientifically sound basis for constructing a trial design that adjusts adaptively for interim tweaks. This modeling strategy may be applied generally to any model‐based early‐phase clinical trial with interim tweaks of the cell therapy being studied.

To make the general strategy concrete, we focus on a model‐based phase 1‐2 trial that uses a joint utility of binary toxicity and binary efficacy to select doses. Since raw dose values $d_{1},\ldots ,d_{J}$ may be on the order of $10^{6}$ cells, and our model parameterization depends on numerical dose values, we use standardized doses $x_{j}=\left\{\log_{10}\left(d_{j}\right)-\log_{10}\left(d_{1}\right)\right\}/\left\{\log_{10}\left(d_{J}\right)-\log_{10}\left(d_{1}\right)\right\}$ for the dose–response model. Alternative standardization methods may be employed, but we recommend that standardized doses should be scaled to take on values between 0 and 1. While, in practice, the investigators may change the set of doses being studied along with the manufacturing process in each stage, to focus on the problem of accounting for stage effects we assume that the set of doses is fixed throughout, as $\left\{x_{1},\ldots ,x_{J}\right\}.$ In fact, our method can accommodate scenarios in which new doses are introduced after a tweak by defining $x_{1},\ldots ,x_{J}$ as the pooled set of doses evaluated both before and after the tweak.

Given maximum overall sample size N, index the first n≤N patients enrolled in the trial by i=1,⋯,n. For the $i^{\mathrm{th}}$ patient, denote their standardized dose by $x_{\left[i\right]}$, their toxicity indicator by $Y_{i}^{T}$, and efficacy indicator by $Y_{i}^{E}$, with $Y_{i}$ = $\left(Y_{i}^{T},Y_{i}^{E}\right).$ To induce association between $Y_{i}^{T}$ and $Y_{i}^{E}$ and account for variability not explained by dose and stage, we define *independent and identically distributed* (*iid*) patient frailties $\varepsilon_{1},\cdots ,\varepsilon_{N}$, and assume that these follow a $N\left(0,\sigma_{\varepsilon }^{2}\right)$ distribution, with probability density function denoted by $\phi \left(\epsilon ,\sigma_{\varepsilon }^{2}\right)$. The variance of the patient frailty distribution $\sigma_{\varepsilon }^{2}$ is unknown and is estimated based on the trial data.

To specify the likelihood, we denote the parameters associated with the probability model for outcome ℓ = T,E in stage k by $\theta_{k}^{\ell }$, with $\theta_{k}=\left(\theta_{k}^{T},\theta_{k}^{E}\right)$. For each dose $x_{j}$, stage k, and patient i, the marginal outcome probabilities given $\varepsilon_{i}$ and $\theta_{k}$ are

$$\pi_{k,i}^{\ell }\left(x_{j},\varepsilon_{i},\theta_{k}^{\ell }\right)=\mathrm{Pr}\left(Y_{i}^{\ell }=1|x_{\left[i\right]}=x_{j},k,\varepsilon_{i},\theta_{k}^{\ell }\right),\;\text{for}\;\ell =T,\;E,$$

and averaging over $\phi \left(\epsilon_{i},\sigma_{\epsilon }^{2}\right)$ gives the unconditional marginals

$$\pi_{k,•}^{\ell }\left(x_{j},\theta_{k}^{\ell }\right)=\int_{\epsilon_{i}}\pi_{k,i}^{\ell }\left(x_{j},\epsilon_{i},\theta_{k}^{\ell }\right)\phi \left(\epsilon_{i},\sigma_{\varepsilon }^{2}\right)d\epsilon_{i}\;\text{for}\;\ell =T,\;E.$$

Denoting possible outcome pairs by y = $\left(y^{T},y^{E}\right)\in \left\{\left(1,0\right),\left(0,1\right),\left(0,0\right),\left(1,1\right)\right\},$ unconditional joint outcome probabilities are computed similarly as

$$\begin{array}{cc} & \mathrm{Pr}\left\{Y_{i}=y|x_{\left[i\right]}=x_{j},k,\theta_{k}\right\}=\int_{\epsilon_{i}}\;\prod_{\ell =T,E}\;{\left\{\pi_{k,i}^{\ell }\left(x_{j},\epsilon_{i},\theta_{k}^{\ell }\right)\right\}}^{y^{\ell }}\\ & {\left\{1-\pi_{k,i}^{\ell }\left(x_{j},\epsilon_{i},\theta_{k}^{\ell }\right)\right\}}^{1-y^{\ell }}\;\phi \left(\epsilon_{i},\sigma_{\epsilon }^{2}\right)d\epsilon_{i}. \end{array}$$

Thus, $\varepsilon_{i}$ induces association between $Y_{i}^{T}$ and $Y_{i}^{E},$ and due to the autoregressive model's induced associations among $\theta_{1},\cdots ,\theta_{K}$, the posterior distributions are correlated across stages.

For functional forms of the marginal outcome probabilities, we assume that the probability of toxicity in the $k^{\mathrm{th}}$ stage given $\varepsilon_{i}$ follows a linear logistic model,

$$\text{logit}\left\{\pi_{k,i}^{T}\left(x_{j},\varepsilon_{i},\theta_{k}^{T}\right)\right\}=\beta_{0k}+e^{\beta_{1k}}x_{j}+\varepsilon_{i},$$

where $\beta_{0k}$ is the baseline toxicity effect in stage k, and the coefficient $e^{\beta_{1k}}$ ensures that the toxicity probability is monotone increasing with dose. We assume that the marginal stage k probability of efficacy given $\varepsilon_{i}$ follows a change‐point logistic regression model,

$$\text{logit}\left\{\pi_{k,i}^{E}\left(x_{j},\varepsilon_{i},\theta_{k}^{E}\right)\right\}=\gamma_{0k}+e^{\gamma_{1k}}x_{j}+\gamma_{2k}{\left(x_{j}-\tau_{k}\right)}_{+}+\gamma_{3k}x_{j}^{2}+\varepsilon_{i},$$

where $u_{+}=\max\left\{u,0\right\}.$ Here, $\gamma_{0k}$ is the baseline log‐odds of efficacy, and $e^{\gamma_{1k}}$ is the linear dose effect, enforcing a monotonic relationship at lower doses. The change‐point term $\gamma_{2k}{\left(x_{j}-\tau_{k}\right)}_{+}$ allows a shift in the dose–response relationship beyond a threshold dose $\tau_{k}$. We assume this to accommodate scenarios where the efficacy probability may decrease or plateau after reaching a certain dose. The quadratic term $\gamma_{3k}x_{j}^{2}$ provides additional flexibility to accommodate non‐linear patterns, such as saturation at an interim dose or decreasing efficacy at higher doses.

**FIGURE 1:**
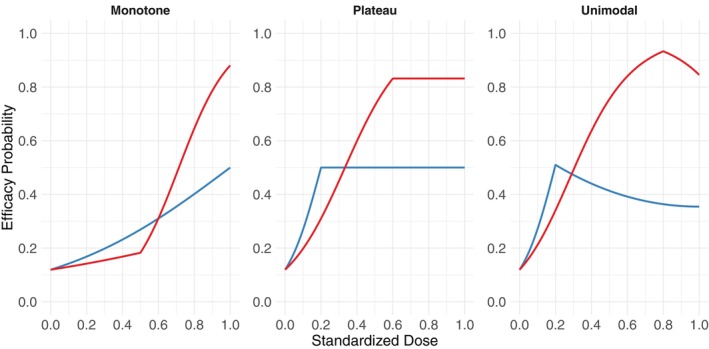
Illustration of possible shapes of the dose‐efficacy curve under change‐point logistic regression model.

Figure 1 illustrates the ability of the efficacy model to represent a broad spectrum of efficacy response profiles, including monotone increasing, a curve that reaches a plateau at an interim dose and does not increase substantially thereafter, and unimodal patterns. This allows the model to characterize a wide range of complex dose–response relationships that may be encountered in an early‐phase clinical trial. Numerical parameter values giving these six curves are provided in the Supporting Information. More flexible dose–response specifications (e.g., polynomial or spline‐based models) could also be incorporated within the proposed BAR12 framework; however, we do not encourage exploring additional model complexity, as the current efficacy formulation strikes a reasonable balance between parsimony and flexibility. Introducing additional parameters may increase estimation variance and lead to poorer operating performance in small early‐phase trials [19].

### 2.2 Establishing Priors

Collecting terms, we denote $\theta_{k}^{T}$ = $\left(\beta_{0k},\beta_{1k}\right)$ and $\theta_{k}^{E}$ = $\left(\gamma_{0k},\gamma_{1k},\gamma_{2k},\gamma_{3k},\tau_{k}\right)$. At the start of the trial in stage 1, before any data are observed, the prior is $\theta_{1}^{\ell }|\theta_{0}^{\ell }∼N\left(\theta_{0}^{\ell },\sum_{0}^{\ell }\right)$. We assume that $\sum_{0}^{\ell }$ is a diagonal matrix so that the components of $\theta_{1}^{\ell }$ have independent, weakly informative priors. To avoid ill‐behaved posteriors, we truncate the support of $\tau_{k}$ to the interval $\left(l_{\tau },u_{\tau }\right)$. When the study doses are standardized to the interval (0,1), a reasonable specification is $\left(l_{\tau },u_{\tau }\right)$ = (0,1.5).

For each stage k≥2, a spike‐and‐slab prior, $p\left(\theta_{k}^{\ell }|\theta_{k-1}^{\ell },\theta_{0}\right)$, which is a mixture of two multivariate normals, is specified as follows:

$$\theta_{k}^{\ell }|\theta_{k-1}^{\ell },\theta_{0}^{\ell }∼\omega^{\ell }N\left(\theta_{k-1}^{\ell },\sum_{k-1}^{\ell }\right)+\left(1-\omega^{\ell }\right)N\left(\theta_{0}^{\ell },\sum_{0}^{\ell }\right).$$

The weakly informative slab component, $N\left(\theta_{0}^{\ell },\sum_{0}^{\ell }\right)$, serves as a baseline prior, while the informative spike component, $N\left(\theta_{k-1}^{\ell },\sum_{k-1}^{\ell }\right)$, is the posterior of $\theta_{k-1}^{\ell }$ given the observed data from previous manufacturing processes in stages 1 through k−1. The weight $\omega^{\ell }\in \left[0,1\right]$ governs how strongly the stage k prior borrows from the $N\left(\theta_{k-1}^{\ell },\sum_{k-1}^{\ell }\right),$ spike, compared to the slab baseline prior $N\left(\theta_{0}^{\ell },\sum_{0}^{\ell }\right)$. The weight parameter $w^{\ell }$ is assumed to follow a weakly informative Beta prior, such as a Beta(1, 1) or Beta(0.5, 0.5). For the variance of the patient frailty distribution, we assume $\sigma_{\varepsilon }^{2}∼\text{Half}-\text{Cauchy}\left(0,\phi \right)$. We do not allow the patient frailty distribution to vary across stages. Therefore, data from all stages contribute to the estimation of the patient frailty distribution, and hence to the correlation between toxicity and efficacy.

The hyperparameters for the slab component, $N\left(\theta_{0}^{\ell },\sum_{0}^{\ell }\right)$, as well as the prior for $\sigma_{\varepsilon }$, must be prespecified before initiating the trial, since they determine the priors used in stage 1.

First, the slab prior means are elicited based on clinicians' beliefs expressed on the probability scale. For example, in a given clinical setting, toxicity probabilities across the dose range may be believed to lie between 0.00 and 0.50, while efficacy probabilities may range from 0.20 to 0.70. These elicited probabilities are then mapped to the corresponding real‐valued regression parameters following the plug‐in approach described in Neuenschwander et al. [20].

Second, after specifying the prior means, the slab prior variance–covariance matrix is calibrated by tuning its dispersion parameters to satisfy two criteria that ensure the design avoids pathological operating behavior [9]:

1. The effective sample size (ESS) of the priors for the toxicity and efficacy probabilities at each dose is constrained to lie between 0 and 2, based on matching the first two moments to those of a Beta(a,b) distribution, for which ESS ≈a+b [21].
2. The probability that the prior mean toxicity or efficacy probability lies within [0.001,0.999] must be sufficiently large (e.g., >0.70), to avoid placing excessive prior mass near the boundaries.

These ESS and probability constraints yield a well‐behaved operational prior. The ESS requirement prevents the prior from exerting undue influence early in the trial when information is sparse, whereas the probability constraint mitigates the risk that extreme prior beliefs distort model‐based decision making.

Finally, the implied prior mean toxicity and efficacy curves are computed at the actual dose levels, and the resulting patterns are reviewed for clinical plausibility. If they appear inconsistent with expert judgment, the elicited inputs or covariance scaling are revised, and the calibration procedure is repeated.

An illustration of how to specify the hyperparameters is as follows. Suppose that the standardized doses are x = (0.0, 0.2, 0.4, 0.6, 0.8, 1.0). Patient‐level frailties are assumed to follow a normal distribution with mean 0 and random variance following a truncated Cauchy hyper‐prior with scale parameter 1. Suppose that, after discussion with the clinicians, the expected probability ranges for toxicity and efficacy are approximately 0.0 to 0.50 and 0.20 to 0.70, respectively. In the first stage, the toxicity probability model's priors are $\beta_{0}∼N\left(-3,{\sqrt{5}}^{2}\right)$ and $\beta_{1}∼N\left(1.1,{\sqrt{2}}^{2}\right)$. The resulting plug‐in prior means for the stage 1 unconditional toxicity probability at the six doses are (0.047,0.083,0.142,0.232,0.355,0.501). The prior ESS values at the doses are (1.466, 0.776, 0.590, 0.520, 0.486, 0.468), which are in the range [0, 2], satisfying the first criterion. The prior probabilities that the toxicity probabilities for the six doses are between 0.001 and 0.999 are (0.923, 0.923, 0.884, 0.842, 0.802, 0.765), exceeding the threshold 0.70, thus fulfilling the second criterion. For the efficacy model, the prior distributions are specified as follows: $\gamma_{0}∼N\left(-1,6\right)$, $\gamma_{1}∼N\left(1.1,2\right)$, $\gamma_{2}∼N\left(-5,3\right)$, and $\gamma_{3}∼N\left(-0.1,2\right)$. The prior for the change point parameter $\tau_{1}$ is a normal distribution truncated to the interval [0, 1.5], denoted by $\tau_{1}∼N_{\left[0,1.5\right]}\left(0.8,1\right)$. The plug‐in prior means for the stage 1 unconditional efficacy probability at the six doses are (0.269,0.401,0.546,0.683,0.792,0.712). These priors are chosen to reflect reasonable efficacy behavior across the dose levels, with prior ESS values (0.993,0.746,0.627,0.553,0.490,0.437). The prior probabilities that the efficacy probabilities for the six doses are between 0.001 and 0.999 are (0.963,0.927,0.868,0.814,0.769,0.728), so these hyperparameters satisfy the two criteria. The prior means and variances of the induced toxicity and efficacy probabilities are provided in the Supporting Information, which show that these induced probabilities lie within a clinically plausible range. For subsequent stages (k≥2), the hyperparameters for the Beta prior on the mixing weights are set to $a_{\omega }=1=b_{\omega }=1$, giving a uniform distribution on [0,1].

## 3 Trial Design

Denote the sample sizes of the successive stages by $n_{1},\cdots ,n_{K}$, and the data obtained during stage k by

$$D^{\left(k\right)}=\left\{\left(Y_{i}^{T},Y_{i}^{E},x_{\left[i\right]}\right),i=\sum_{r=1}^{k-1}n_{r}+1,\ldots ,\sum_{r=1}^{k}n_{r}\right\}.$$

Let $D_{n}$ denote the accumulated data up to the $n^{\mathrm{th}}$ patient, regardless of stage. For example, if two manufacturing processes have been used up to patient n, then $D_{n}=D^{\left(1\right)}\cup D^{\left(2\right)}$.

### 3.1 Dose Acceptability

As in Thall and Russell [7] and many others, let ${\bar{\pi }}^{T}$ denote a specified fixed upper limit on all $\pi_{k,•}^{T}\left(x_{j},\theta_{k}^{T}\right)$ and ${\underline{\pi }}^{E}$ a specified fixed lower limit on all $\pi_{k,•}^{E}\left(x_{j},\theta_{k}^{E}\right).$ These values should be elicited from the physicians planning the trial. Given observed data $D_{n}$ during the $k^{\mathrm{th}}$ stage, a standardized dose x of cells obtained from the $k^{\mathrm{th}}$ manufacturing process is considered *acceptable* if it has a sufficiently low posterior probability of excessive toxicity and a sufficiently high posterior probability of efficacy. Formally, dose x is acceptable if

(1) $$\mathrm{Pr}\left\{\pi_{k,•}^{T}\left(x,\theta_{k}^{T}\right)>{\bar{\pi }}^{T}|D_{n}\right\}<c^{T}\;\text{and}\;\mathrm{Pr}\left\{\pi_{k,•}^{E}\left(x,\theta_{k}^{E}\right)>{\underline{\pi }}^{E}|D_{n}\right\}>c^{E},$$

where $c^{T}$ and $c^{E}$ are pre‐specified probability cutoffs. These cutoffs should be calibrated to obtain large probabilities of declaring x unacceptable if $\pi_{k,•}^{T}{\left(x\right)}^{\text{true}}$ is substantially larger than ${\bar{\pi }}^{T}$ or $\pi_{k,•}^{E}{\left(x\right)}^{\text{true}}$ is substantially smaller than ${\underline{\pi }}^{E}$. Based on practical and clinical considerations, unacceptable doses will not be reconsidered within the same stage. However, such a dose may be reconsidered after a manufacturing tweak, because a tweak may improve its safety or efficacy. Denote the set of acceptable doses using cells created with the $k^{\mathrm{th}}$ manufacturing process by $A_{n,k}.$.

### 3.2 Utilities

Identifying an optimal dose for each patient cohort is based on a utility, as in Thall and Nguyen [22], Lin et al. [11], and Lee et al. [16]. To reduce notation, temporarily suppress the indices j, k, and i. Let U(y) denote the elicited utility of each possible joint outcome pair y = $\left(y^{T},y^{E}\right)$. The utility of the best possible joint outcome, (no toxicity with response) = (0,1), may be set to U(0,1)=100, and the worst possible outcome, (toxicity with no response) = (1,0), assigned U(1,0)=0. The utilities for the remaining intermediate outcomes then are elicited subject to the consistency constraints 0<U(0,0)<100 and 0<U(1,1)<100. The *mean utility* of administering a standardized dose x to a patient, using cells produced by the k‐th manufacturing process, is defined as

$$\bar{U}\left(x,k,\theta_{k}\right)=\sum_{y^{T}=0}^{1}\sum_{y^{E}=0}^{1}U\left(y^{T},y^{E}\right)\mathrm{Pr}\left(Y^{T}=y^{T},Y^{E}=y^{E}|x,k,\theta_{k}\right).$$

From a decision‐theoretic perspective, $\theta_{k}$ is the *state of nature* and x is the *action*. Let $p\left(\theta_{k}|D_{n}\right)$ denote the posterior distribution of $\theta_{k}$ given data $D_{n}$. The optimal dose in stage k is defined as that maximizing the *posterior mean utility*, given by

$$u\left(x,k,D_{n}\right)=\int \bar{U}\left(x,k,\theta_{k}\right)\;p\left(\theta_{k}|D_{n}\right)d\theta_{k},$$

which quantifies the desirability of x based on the current data $D_{n}$.

### 3.3 Trial Conduct

In addition to establishing fixed prior hyperparameters, design parameters that must be specified include the maximum overall sample size N, cohort size c, acceptability limits ${\bar{\pi }}^{T}$ and ${\underline{\pi }}^{E}$, probability cutoffs $c^{T}$ and $c^{E}$. The first cohort is treated at a starting dose chosen by the physicians. For each subsequent cohort, given the accumulated data $D_{n}$ during manufacturing stage k, the posterior decision criteria are evaluated. If An,k=0 then the trial is terminated without recommending a dose, otherwise a dose is selected from $A_{n,k}$ to maximize $u\left(x,k,D_{n}\right)$, subject to the constraint that an untried dose may not be skipped when escalating. This is continued until the maximum sample size N is reached or no dose is acceptable. Details of the dose‐finding algorithm are provided below.

Conventionally, two designs typically are used for dealing with tweaks, and these may be considered competitors to the BAR12 design. The first, referred to as the “Identical” design, assumes that the toxicity and efficacy probability distributions are the same across different manufacturing processes, so this design ignores all tweaks. The “Separate” design treats the manufacturing processes as yielding entirely different distributions for toxicity and efficacy, so data from stages prior to a tweak may not be used when making later decisions. In contrast with these approaches, BAR12 uses the decision criteria $A_{n,k}$ and $u\left(x,k,D_{n}\right)$, derived under the Bayesian autoregressive model that accounts for stage‐specific effects introduced by tweaks, and borrows information across stages.

Consider a phase 1‐2 trial with standardized doses $\left\{x_{1},\cdots ,x_{J}\right\}$ fixed throughout, cohort size c, interim number of best doses to study L, and maximum sample sizes N. The dose finding algorithm is as follows:

Step 1. At the start of stage 1, treat the first cohort at a starting dose chosen by the physician.

Step 2. For each new cohort when n patients have been treated:

- *Stop if Needed*: If An,1=0, terminate the trial and do not select any dose.
- *Choose the Cohort's Dose*: If An,1≠0, treat the next cohort at the dose $x\in A_{n,1}$ with highest posterior mean utility $u\left(x,1,D_{n}\right)$, subject to the constraint that an untried dose may not be skipped when escalating.
- If there is no tweak, repeat 2(a) and 2(b) until $n_{1}=N$ patients have been treated and choose the best final dose from $A_{N,1}$ to maximize $u\left(x,1,D_{N}\right)$. If there is a tweak before N patients have been treated, go to Step 3.

Step 3. Actions for each Stage k≥2 when n patients have been treated:

- *Stop if Needed*: If An,k=0, terminate the trial and do not select any dose.
- *Identify* L *Candidate Doses*: If An,k≠0, select L doses with the highest posterior mean utilities $u\left(x,k,D_{n}\right)$ among the doses in $A_{n,k}$.
- *Adaptively Randomize Patients*: For each cohort of c×L patients in stage k, randomize patients among the best L doses in $A_{n,k}$, with c patients per dose, using the adaptive randomization (AR) probabilities specified in (2).
- If there is no new tweak, repeat 3(a–c) until N patients have been treated and choose the best final dose from $A_{N,k}$ to maximize $u\left(x,k,D_{N}\right)$. If there is a new tweak before N patients have been treated, repeat step 3 for stage k+1.

For k≥2 stages, both the “Identical” and “Separate” designs assume a weakly informative prior and do not make any model changes. In contrast, the BAR12 design assumes a weakly‐informative prior in stage 1, but for each stage k≥2 it applies the spike‐and‐slab prior that incorporates data from the current stage and previous stages.

At the start of Stage k>1, because there are no data for the new manufacturing process, the optimal dose from Stage k−1 provides the most reliable reference. Given that a tweak is expected to cause at most moderate changes, we assume that the optimal dose in stage k is likely to be similar to its predecessor in stage k−1. To accommodate potential differences between stages, following a tweak, BAR12 employs AR among the L candidate doses from the prior stage's admissible set $A_{n,k-1}$ having the highest posterior mean utilities. In practice, L = 2 or 3 is feasible in step 3. The AR probabilities are proportional to their posterior mean utilities, and the set of L doses is updated iteratively based on the current admissible set $A_{n,k}$. This balances the assumption of dose similarity across trial stages with the ability to identify an optimal dose based on the trial data.

To implement, the AR in stage k, the L admissible doses with the highest posterior mean utilities are selected to form the candidate dose set,

$$C_{k,n}=\left\{\left\{x_{\left(1\right)},\ldots ,x_{\left(L\right)}\right\}:\;u\left(x_{\left(1\right)},k,D_{n}\right)\geq \cdots \geq u\left(x_{\left(L\right)},k,D_{n}\right)\right\},$$

where the doses in $C_{k,n}$ may change as new data are obtained. Each successive block of c×L patients are randomized among the doses in $C_{k,n}$ using the probabilities

(2) $$p_{k}\left(x_{\left(\ell \right)}|D_{n}\right)=\frac{u{\left(x_{\left(\ell \right)},k,D_{n}\right)}^{\kappa }}{\sum_{x\in C_{k,n}}u{\left(x,k,D_{n}\right)}^{\kappa }},$$

where κ≥0 is a tuning parameter that controls the degree of preference for higher‐utility doses. Larger values of κ yield more aggressive allocation to the highest‐utility dose, while smaller values promote greater exploration. In particular, setting κ=0 corresponds to equal randomization. This procedure is repeated for each cohort until the maximum sample size N is reached, a tweak moves the trial from stage k to stage k+1, or no admissible doses remain. This approach addresses the well known “optimization versus exploration” dilemma in sequential decision making [23] by assigning a greater proportion of patients to doses with higher expected utility, while still allowing exploration of all admissible doses. In contrast, a greedy algorithm that repeatedly chooses the dose x maximizing $u\left(x,k,D_{n}\right)$ risks getting stuck at a suboptimal dose.

## 4 Simulation Study

### 4.1 Simulation Design

Ten simulation scenarios were constructed to assess OCs of the BAR12 design and compare it to the “Identical” and “Separate” designs described in Section 3.3. Six raw dose values $\left\{1,2.5,6.3,15.8,39.8,100\right\}\times 10^{6}$ were considered, corresponding to standardized doses x∈ {0.0, 0.2, 0.4, 0.6, 0.8, 1.0}. The cohort size was c=3. The clinical acceptability limits were ${\bar{\pi }}^{T}=0.30$ and ${\underline{\pi }}^{E}=0.20,$ with the dose acceptability cutoffs $c^{T}=0.70$ for toxicity and $c^{E}=0.85$ for efficacy. In stage 2, AR was implemented using a selection set size of L=2 and tuning parameter κ=1. A maximum of 42 patients were enrolled, with 18 patients enrolled prior to the manufacturing tweak (stage 1) and 24 patients afterward (stage 2), denoted as 18/24 (*N* = 42). In addition to this primary 18/24 sample size allocation, the design's behavior was evaluated under the alternative splits 24/24 and 18/36. The results are presented in Section 5. Clinical utilities of the intermediate outcomes were set to U(1,0)=0, U(0,0)=40, U(1,1)=60, and U(0,1)=100. We adopted the same prior specification as presented in Section 2.2 for all three designs.

**FIGURE 2:**
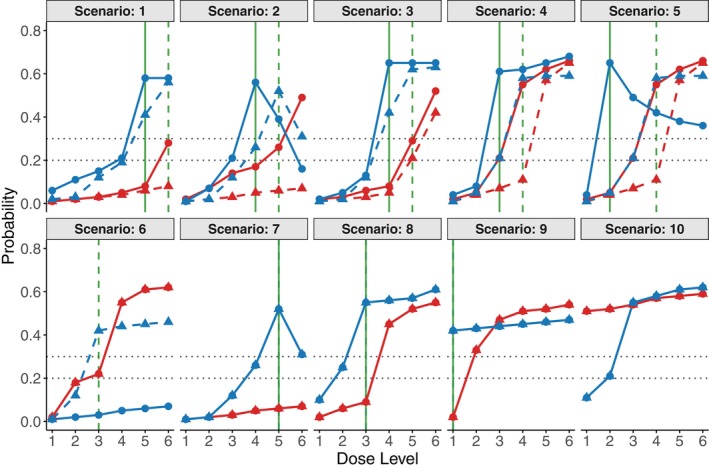
True dose–response relationships assumed for the simulation scenarios. The red and blue curves represent the true probabilities of toxicity ( $\pi^{T}$ ) and efficacy ( $\pi^{E}$ ), respectively. Dashed lines correspond to manufacturing process 1, while solid lines correspond to manufacturing process 2 after the tweak. The green dashed and solid vertical lines identify the true optimal doses for processes 1 and 2, respectively. Horizontal dotted lines identify the upper toxicity probability limit ${\bar{\pi }}^{T}=0.3$ and the minimum clinically acceptable efficacy probability ${\underline{\pi }}^{E}=0.2$.

For convenience when describing the simulations, we will refer to the doses in terms of their levels, 1,…, 6. A total of ten scenarios were examined, in which the true toxicity and efficacy rates were specified to reflect real‐world conditions, rather than being generated from the assumed outcome model (i.e., scenarios involving model misspecification). The assumed true dose–response relationships are illustrated in Figure 2. In scenarios 1 to 6, the dose‐toxicity and dose‐efficacy curves change substantially after the tweak. For example, in scenario 1, the true dose‐efficacy curve is monotone increasing in stage 1 but transitions to a unimodal shape that initially increases and then decreases in stage 2. Scenario 5 provides another example, illustrating a change from a plateau efficacy curve in stage 1 to a unimodal curve in stage 2. Consequently, scenarios 1 to 6 have respective optimal stage 1 dose levels 6, 5, 5, 4, 4, and 3, and optimal stage 2 dose levels 5, 4, 4, 3, 2, and 0 after the tweak, where the value “0” denotes a scenario where there is no optimal dose level for that stage. In scenarios 7 to 10, the pre‐tweak and post‐tweak stages have identical dose‐toxicity and dose‐efficacy curves. The optimal dose levels in the remaining four scenarios are 5, 3, 1, and 0, respectively.

**FIGURE 3:**
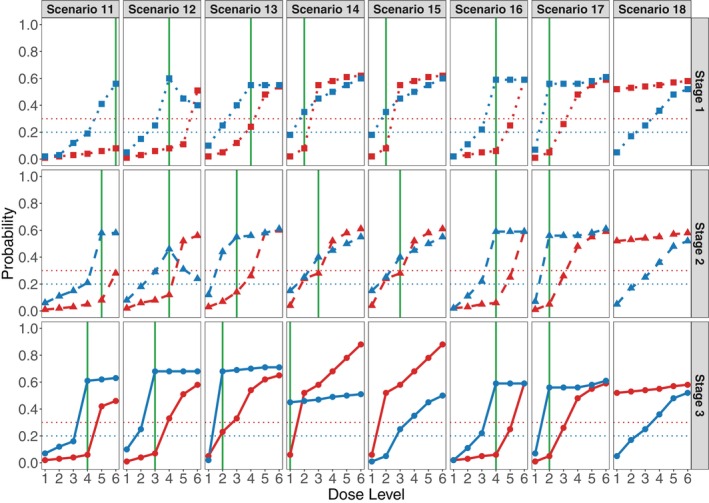
True dose–response relationships for the simulation scenarios. The red and blue curves represent true probabilities of toxicity ( $\pi^{T}$ ) and efficacy ( $\pi^{E}$ ), respectively. Dotted, dashed, and solid lines correspond to manufacturing stages 1, 2, and 3. The green lines identify true optimal doses for processes 1, 2, and 3, shown separately in each plot. Horizontal dotted lines at 0.30 and 0.20 denote the upper toxicity threshold (${\bar{\pi }}^{T}=0.3$) and the minimum clinically acceptable efficacy level (${\underline{\pi }}^{E}=0.2$), respectively.

We also examined more complex settings with three stages, due to two manufacturing tweaks, based on eight scenarios (i.e., scenarios 11–18). The true dose–response relationships for these scenarios are illustrated in Figure 3. In these scenarios, the dose‐toxicity and dose‐efficacy relationships were designed to change across the three stages, in a variety of different ways. In the heterogeneous scenarios 11 to 15, the optimal dose varies between stages due to changing dose–response profiles. The respective true optimal doses in stage 1 are 6, 4, 4, 2, and 2. After the first tweak, the optimal doses in stage 2 are shifted to 5, 4, 3, 3, and 3. After the second tweak, the optimal doses in stage 3 are 4, 3, 2, 1, and 0. In contrast, for the homogeneous scenarios 16, 17, and 18, the true optimal doses are constant across the three stages and are 4, 2, and 0, respectively. For these scenarios, we assumed a total of up to 54 patients, with 15 patients in stage 1 prior to the first manufacturing tweak, 15 in stage 2, and 24 in stage 3 following the second tweak.

### 4.2 Simulation Results

Table 1 summarizes the OCs for trials with a manufacturing change and a maximum of 42 patients, based on 1000 simulation replications. In scenarios 1 to 6, which have moderate heterogeneity between the two stages, the BAR12 design has the best OCs compared to the Identical and Separate designs, with a much larger probability of selecting the optimal dose. In Scenarios 7–10, where the dose–response relationships are unchanged following the tweak, the Identical design has slightly better performance than the BAR12 design, as may be expected, but the BAR12 design consistently outperforms the Separate design. This superior performance may be attributed to BAR12's ability to borrow information across different manufacturing processes efficiently when there is no heterogeneity, thereby enhancing the probability of correctly identifying the true optimal dose after the last tweak.

**TABLE 1:** Simulation results for the BAR12, Identical, and Separate designs, under Scenarios 1–10, with 18 patients in stage 1 and 24 patients in stage 2 (denoted 18/24).

True parameter values							Designs							
Dose	Stage 1			Stage 2			BAR12			Identical			Separate	
Level	$\pi_{1}^{T}$	$\pi_{1}^{E}$	$U_{1}$	$\pi_{2}^{T}$	$\pi_{2}^{E}$	$U_{2}$	$n_{1}$	$n_{2}$	Sel	$n_{1}$	$n_{2}$	Sel	$n_{2}$	Sel
**Scenario 1**														
1	*0.01*	*0.02*	*40.8*	*0.01*	*0.06*	*43.2*	3.0	0.1	0.0	3.0	0.0	0.0	3.0	0.0
2	*0.02*	*0.03*	*41.0*	*0.02*	*0.11*	*45.8*	3.0	0.2	0.4	3.0	0.2	0.4	3.1	1.9
3	*0.03*	*0.12*	*46.0*	*0.03*	*0.15*	*47.8*	3.0	0.9	2.1	3.0	0.4	1.6	3.2	5.7
4	*0.04*	*0.19*	*49.8*	*0.05*	*0.21*	*50.6*	3.0	4.3	10.5	3.0	1.3	5.6	3.6	11.5
5	0.06	0.41	62.2	**0.08**	**0.58**	**71.6**	**3.0**	**10.6**	**57.0**	**3.0**	**4.4**	**26.8**	**4.3**	**28.8**
6	**0.08**	**0.56**	**70.4**	0.28	0.58	63.6	3.0	7.4	27.6	3.0	16.9	60.8	6.6	49.9
0	0.00	0.00	0.0	0.00	0.00	0.0	17.9	23.5	2.4	17.9	23.3	4.8	23.8	2.2
**Scenario 2**														
1	*0.01*	*0.01*	*40.2*	*0.02*	*0.01*	*39.8*	3.0	0.2	0.7	3.0	0.0	0.0	3.1	1.0
2	*0.02*	*0.02*	*40.4*	*0.07*	*0.07*	*41.4*	3.0	1.3	4.9	3.0	0.1	0.0	3.8	10.6
3	*0.03*	*0.12*	*46.0*	*0.14*	*0.21*	*47.0*	3.0	5.2	23.9	3.0	1.2	5.2	4.5	23.7
4	0.05	0.26	53.6	**0.17**	**0.56**	**66.8**	**3.0**	**8.1**	**57.8**	**3.0**	**8.6**	**48.3**	**5.0**	**43.8**
5	**0.06**	**0.52**	**68.8**	0.26	0.39	53.0	3.0	6.0	9.2	3.0	8.8	37.1	3.8	8.4
6	0.07	0.31	55.8	*0.49*	*0.16*	*30.0*	3.0	2.6	0.1	3.0	4.0	1.1	2.7	0.9
0	0.00	0.00	0.0	0.00	0.00	0.0	18.0	23.4	3.4	18.0	22.7	8.3	22.9	11.6
**Scenario 3**														
1	*0.01*	*0.01*	*40.2*	*0.02*	*0.02*	*40.4*	3.0	0.1	0.0	3.0	0.0	0.0	3.0	0.1
2	*0.02*	*0.02*	*40.4*	*0.03*	*0.05*	*41.8*	3.0	0.5	0.7	3.0	0.1	0.1	3.1	0.9
3	*0.03*	*0.12*	*46.0*	*0.06*	*0.13*	*45.4*	3.0	3.3	7.0	3.0	0.7	2.4	3.4	5.7
4	0.05	0.42	63.2	**0.08**	**0.65**	**75.8**	**3.1**	**8.1**	**54.0**	**3.1**	**7.4**	**42.5**	**4.9**	**38.9**
5	**0.21**	**0.62**	**68.8**	0.29	0.65	67.4	3.0	7.9	32.3	3.0	9.8	46.4	4.7	33.5
6	*0.42*	*0.63*	*61.0*	*0.52*	*0.65*	*58.2*	2.8	3.6	3.5	2.8	4.8	1.9	4.5	16.6
0	0.00	0.00	0.0	0.00	0.00	0.0	17.9	23.4	2.5	17.9	22.7	6.7	23.5	4.3
**Scenario 4**														
1	*0.01*	*0.01*	*40.2*	*0.02*	*0.04*	*41.6*	3.0	1.5	2.3	3.0	0.0	0.1	3.3	1.8
2	*0.04*	*0.05*	*41.4*	*0.05*	*0.08*	*42.8*	3.1	5.8	24.4	3.1	1.0	3.7	6.3	29.8
3	0.07	0.21	49.8	**0.21**	**0.61**	**68.2**	**3.3**	**7.4**	**55.4**	**3.3**	**8.9**	**53.9**	**7.4**	**47.1**
4	**0.11**	**0.58**	**70.4**	*0.55*	*0.62*	*55.2*	3.4	4.7	6.9	3.4	6.5	6.4	3.8	9.4
5	*0.57*	*0.59*	*52.6*	*0.62*	*0.65*	*54.2*	2.9	1.8	0.4	2.9	1.6	1.0	1.8	2.1
6	*0.65*	*0.59*	*49.4*	*0.66*	*0.68*	*54.4*	1.9	0.6	0.0	1.9	0.6	0.0	0.8	0.6
0	0.00	0.00	0.0	0.00	0.00	0.0	17.6	21.8	10.6	17.6	18.7	34.9	23.4	9.2
**Scenario 5**														
1	*0.01*	*0.01*	*40.2*	*0.02*	*0.04*	*41.6*	3.1	2.2	2.8	3.1	0.0	0.0	3.7	5.5
2	*0.04*	*0.05*	*41.4*	***0.05***	***0.65***	***77.0***	**3.1**	**6.5**	**58.9**	**3.1**	**1.6**	**9.0**	**7.1**	**67.0**
3	0.07	0.21	49.8	0.21	0.49	61.0	3.3	7.2	27.5	3.3	9.3	52.6	7.2	23.3
4	**0.11**	**0.58**	**70.4**	*0.55*	*0.42*	*43.2*	3.4	4.2	2.9	3.4	6.8	9.2	3.4	1.4
5	*0.57*	*0.59*	*52.6*	*0.62*	*0.38*	*38.0*	2.9	1.6	0.3	2.9	1.4	0.6	1.7	0.2
6	*0.65*	*0.59*	*49.4*	*0.66*	*0.36*	*35.2*	2.0	0.5	0.0	2.0	0.5	0.0	0.6	0.2
0	0.00	0.00	0.0	0.00	0.00	0.0	17.7	22.2	7.6	17.7	19.5	28.6	23.7	2.4
**Scenario 6**														
1	*0.02*	*0.01*	*39.8*	*0.02*	*0.01*	*39.8*	3.9	2.6	0.6	3.9	0.4	0.0	3.4	0.6
2	*0.18*	*0.12*	*40.0*	*0.18*	*0.02*	*34.0*	4.8	4.4	3.0	4.8	5.8	7.8	3.5	1.0
3	0.22	0.42	56.4	*0.22*	*0.03*	*33.0*	3.5	4.1	6.6	3.5	6.5	22.0	2.8	0.7
4	*0.55*	*0.44*	*44.4*	*0.55*	*0.05*	*21.0*	2.6	1.9	3.6	2.6	0.9	1.4	2.4	0.4
5	*0.61*	*0.45*	*42.6*	*0.61*	*0.06*	*19.2*	1.3	0.6	0.1	1.3	0.1	0.2	1.1	0.3
6	*0.62*	*0.46*	*42.8*	*0.62*	*0.07*	*19.4*	0.5	0.2	0.1	0.5	0.0	0.1	0.4	0.0
0	**0.00**	**0.00**	**0.0**	**0.00**	**0.00**	**0.0**	**16.5**	**13.8**	**86.0**	**16.5**	**13.8**	**68.5**	**13.6**	**97.0**
**Scenario 7**														
1	*0.01*	*0.01*	*40.2*	*0.01*	*0.01*	*40.2*	3.0	0.1	0.0	3.0	0.0	0.0	3.0	0.0
2	*0.02*	*0.02*	*40.4*	*0.02*	*0.02*	*40.4*	3.0	0.2	0.1	3.0	0.1	0.0	3.0	0.3
3	*0.03*	*0.12*	*46.0*	*0.03*	*0.12*	*46.0*	3.0	1.1	2.0	3.0	0.9	2.8	3.1	2.8
4	0.05	0.26	53.6	0.05	0.26	53.6	3.0	5.9	14.6	3.0	3.0	11.5	3.5	9.8
5	**0.06**	**0.52**	**68.8**	**0.06**	**0.52**	**68.8**	**3.0**	**10.7**	**68.3**	**3.0**	**7.3**	**41.5**	**4.1**	**28.7**
6	0.07	0.31	55.8	0.07	0.31	55.8	3.0	5.7	12.5	3.0	11.9	39.9	7.0	54.5
0	0.00	0.00	0.0	0.00	0.00	0.0	18.0	23.5	2.5	18.0	23.2	4.3	23.7	3.9
**Scenario 8**														
1	*0.02*	*0.10*	*45.2*	*0.02*	*0.10*	*45.2*	3.4	1.5	2.0	3.4	0.8	2.4	3.3	2.7
2	0.06	0.25	52.6	0.06	0.25	52.6	4.1	6.0	17.0	4.1	6.1	24.9	4.4	19.7
3	**0.09**	**0.55**	**69.4**	**0.09**	**0.55**	**69.4**	**3.9**	**8.8**	**57.5**	**3.9**	**12.1**	**60.2**	**6.9**	**51.0**
4	*0.45*	*0.56*	*55.6*	*0.45*	*0.56*	*55.6*	3.1	5.3	18.5	3.1	3.1	7.8	4.7	17.6
5	*0.52*	*0.57*	*53.4*	*0.52*	*0.57*	*53.4*	2.2	1.4	2.1	2.2	0.9	1.0	2.9	5.2
6	*0.55*	*0.61*	*54.6*	*0.55*	*0.61*	*54.6*	1.2	0.3	0.1	1.2	0.2	0.0	1.5	1.6
0	0.00	0.00	0.0	0.00	0.00	0.0	17.9	23.4	2.8	17.9	23.2	3.7	23.8	2.2
**Scenario 9**														
1	**0.02**	**0.42**	**64.4**	**0.02**	**0.42**	**64.4**	***8.9***	***12.1***	***77.3***	***8.9***	***17.1***	***76.7***	***10.3***	***72.1***
2	0.33	0.43	52.6	0.33	0.43	52.6	*4.1*	*8.8*	*17.3*	*4.1*	*4.7*	*16.4*	*7.5*	*19.3*
3	*0.47*	*0.44*	*47.6*	*0.47*	*0.44*	*47.6*	*2.4*	*2.0*	*2.6*	*2.4*	*1.1*	*3.3*	*3.3*	*4.5*
4	*0.51*	*0.45*	*46.6*	*0.51*	*0.45*	*46.6*	*1.4*	*0.4*	*0.3*	*1.4*	*0.3*	*0.4*	*1.6*	*2.0*
5	*0.52*	*0.46*	*46.8*	*0.52*	*0.46*	*46.8*	*0.7*	*0.1*	*0.0*	*0.7*	*0.1*	*0.1*	*0.8*	*0.2*
6	*0.54*	*0.47*	*46.6*	*0.54*	*0.47*	*46.6*	*0.3*	*0.0*	*0.0*	*0.3*	*0.0*	*0.0*	*0.3*	*0.0*
0	0.00	0.00	0.0	0.00	0.00	0.0	*17.8*	*23.4*	*2.5*	*17.8*	*23.3*	*3.1*	*23.8*	*1.9*
**Scenario 10**														
1	*0.51*	*0.11*	*26.2*	*0.51*	*0.11*	*26.2*	4.7	0.9	0.9	4.7	0.7	1.0	5.1	4.2
2	*0.52*	*0.21*	*31.8*	*0.52*	*0.21*	*31.8*	1.9	0.5	0.3	1.9	0.4	0.5	2.0	2.4
3	*0.54*	*0.55*	*51.4*	*0.54*	*0.55*	*51.4*	0.9	0.2	0.2	0.9	0.2	0.2	0.9	1.6
4	*0.57*	*0.58*	*52.0*	*0.57*	*0.58*	*52.0*	0.4	0.1	0.1	0.4	0.1	0.0	0.3	0.2
5	*0.58*	*0.61*	*53.4*	*0.58*	*0.61*	*53.4*	0.1	0.0	0.0	0.1	0.0	0.0	0.1	0.0
6	*0.59*	*0.62*	*53.6*	*0.59*	*0.62*	*53.6*	0.0	0.0	0.0	0.0	0.0	0.0	0.0	0.1
0	**0.00**	**0.00**	**0.0**	**0.00**	**0.00**	**0.0**	**8.1**	**1.7**	**98.5**	**8.1**	**1.4**	**98.3**	**8.5**	**91.5**

*Note*: For each stage k =1, 2, $\pi_{k}^{T}$, $\pi_{k}^{E}$, and $U_{k}$ are the true toxicity probability, efficacy probability, and mean utility. Sel = selection percentage at each dose. The true optimal dose in each scenario is given in boldface, and unacceptable doses are in italics. $n_{1}$ and $n_{2}$ denote the mean numbers of patients in stages 1 and 2. “0” denotes no dose selected.

In Section S1 of the Supporting Information, we provide comprehensive MCMC diagnostics, including Gelman–Rubin $\hat{R}$ statistics, MCMC ESS, Monte Carlo standard errors (MCSE), and 95% credible intervals (CIs) for all model parameters at the end of Stage 1 and Stage 2 across all 10 scenarios. These summaries are based on 1000 simulated trials, each fitted using four MCMC chains with 10 000 post‐warm‐up draws. Overall, the diagnostics demonstrate stable and reliable MCMC performance. For all regression parameters, the $\hat{R}$ values are consistently close to 1, with large ESS and small MCSE, indicating good mixing and high precision of posterior summaries. The posterior standard deviations and CI widths decrease from Stage 1 to Stage 2, reflecting increased information as more patients are enrolled, particularly in scenarios where the dose‐outcome relationships are consistent across stages. Although the variance parameter $\sigma_{\varepsilon }^{2}$ exhibits relatively smaller ESS and larger MCSE, this behavior is expected in small‐sample hierarchical models and does not affect convergence, as its $\hat{R}$ values remain close to 1 across all scenarios. Taken together, these results confirm that the MCMC chains converge reliably and provide stable posterior inference in both stages of the proposed design.

Similar trial OC patterns are seen in scenarios involving two manufacturing tweaks, given in Table 2. These settings are more complex, since the optimal dose may change across the three stages. For example, in Scenario 11, the optimal dose changes from 5 in Stage 1 to 4 in Stage 2 and to 3 in Stage 3. In this scenario, the BAR12 design achieves a 68.6% correct selection rate, substantially outperforming the Identical and Separate designs, which have respective correct selection rates of 25.5% and 48.3%. In Scenario 12, where the optimal dose remains at 4 in the first two stages and then changes to 3 in Stage 3, BAR12 also performs best, with a 51.6% correct selection rate compared to 35.8% for the Identical design and 50.0% for the Separate design. Similarly, in Scenario 14, the optimal dose changes from 2 to 3 and then to 1. In this scenario, the BAR12 design outperforms the Identical design by more than 20 percentage points. When the optimal dose is level 1, a smaller sample size generally is sufficient for reliable dose selection, which can lead to favorable performance for the Separate design. Nevertheless, in these more complex scenarios with two tweaks, the BAR12 design consistently outperforms both the Identical and Separate designs.

**TABLE 2:** Simulation results for the BAR12, Identical, and Separate designs, under Scenarios 11–18, with 15 patients in stage 1, 15 patients in stage 2, and 24 patients in stage 3 (denoted 15/15/24).

True Scenario Parameters										Designs									
Dose	Stage 1			Stage 2			Stage 3			BAR12				Identical				Separate	
Level	$\pi_{1}^{T}$	$\pi_{1}^{E}$	$U_{1}$	$\pi_{2}^{T}$	$\pi_{2}^{E}$	$U_{2}$	$\pi_{3}^{T}$	$\pi_{3}^{E}$	$U_{3}$	$n_{1}$	$n_{2}$	$n_{3}$	Sel	$n_{1}$	$n_{2}$	$n_{3}$	Sel	$n_{3}$	Sel
**Scenario 11**																			
1	*0.01*	*0.02*	*40.8*	*0.01*	*0.06*	*43.2*	*0.02*	*0.07*	*43.4*	3.0	0.1	0.1	0.2	3.0	0.0	0.0	0.0	3.1	0.7
2	*0.02*	*0.03*	*41.0*	*0.02*	*0.11*	*45.8*	*0.03*	*0.12*	*46.0*	3.0	0.4	0.8	1.6	3.0	0.2	0.1	0.2	3.5	9.7
3	*0.03*	*0.12*	*46.0*	*0.03*	*0.15*	*47.8*	*0.04*	*0.16*	*48.0*	3.0	1.0	5.1	13.2	3.0	0.7	0.9	2.9	4.2	22.8
4	*0.04*	*0.19*	*49.8*	*0.05*	*0.21*	*50.6*	***0.06***	***0.61***	***74.2***	**3.0**	**3.8**	**8.8**	**68.6**	**3.0**	**1.2**	**3.5**	**25.5**	**5.3**	**48.3**
5	0.06	0.41	62.2	**0.08**	**0.58**	**71.6**	*0.42*	*0.42*	*48.4*	3.0	6.2	5.6	7.8	3.0	4.4	11.2	52.9	4.4	11.6
6	**0.08**	**0.56**	**70.4**	0.28	0.58	63.6	*0.46*	*0.31*	*40.2*	0.0	2.4	1.6	0.0	0.0	7.8	6.6	7.4	3.2	3.0
0	0.00	0.00	0.0	0.00	0.00	0.0	0.00	0.00	0.0	15.0	13.9	22.0	8.6	15.0	14.2	22.2	11.1	23.7	3.9
**Scenario 12**																			
1	*0.01*	*0.05*	*42.6*	*0.02*	*0.08*	*44.0*	*0.01*	*0.10*	*45.6*	3.1	0.2	0.5	0.4	3.1	0.1	0.1	0.2	3.1	0.3
2	*0.03*	*0.15*	*47.8*	*0.06*	*0.18*	*48.4*	*0.04*	*0.25*	*53.4*	3.0	1.1	3.4	6.9	3.0	0.3	0.4	1.7	3.9	9.7
3	0.06	0.25	52.6	0.08	0.29	54.2	**0.07**	**0.68**	**78.0**	**3.0**	**4.0**	**9.0**	**51.6**	**3.0**	**3.0**	**6.8**	**35.8**	**6.1**	**50.0**
4	**0.08**	**0.60**	**72.8**	**0.12**	**0.46**	**62.8**	0.33	0.68	67.6	3.0	5.9	8.3	37.0	3.0	6.8	15.4	58.4	5.7	32.5
5	0.11	0.45	62.6	*0.52*	*0.31*	*37.8*	*0.51*	*0.68*	*60.4*	3.0	3.6	2.5	3.4	3.0	3.8	0.7	0.9	3.4	5.9
6	*0.51*	*0.40*	*43.6*	*0.56*	*0.24*	*32.0*	*0.58*	*0.68*	*57.6*	0.0	0.2	0.1	0.0	0.0	0.8	0.1	0.1	1.9	1.1
0	0.00	0.00	0.0	0.00	0.00	0.0	0.00	0.00	0.0	15.0	14.9	23.8	0.7	15.0	14.9	23.5	2.9	24.0	0.5
**Scenario 13**																			
1	*0.02*	*0.10*	*45.2*	*0.03*	*0.12*	*46.0*	*0.05*	*0.02*	*39.2*	3.2	0.7	3.6	4.9	3.1	0.2	0.2	0.8	4.4	5.8
2	0.05	0.25	53.0	0.07	0.44	63.6	**0.15**	**0.68**	**74.8**	**3.2**	**3.1**	**8.1**	**58.4**	**3.2**	**2.7**	**5.4**	**29.7**	**7.6**	**48.3**
3	0.12	0.40	59.2	**0.14**	**0.55**	**67.4**	0.33	0.69	68.2	3.1	4.9	7.4	28.8	3.1	5.8	13.4	60.2	6.2	28.5
4	**0.24**	**0.55**	**63.4**	0.26	0.56	63.2	*0.54*	*0.70*	*60.4*	2.9	4.1	3.7	3.5	2.9	4.3	4.0	5.6	3.0	4.9
5	*0.48*	*0.55*	*53.8*	*0.58*	*0.58*	*51.6*	*0.62*	*0.71*	*57.8*	2.6	1.8	0.5	0.1	2.6	1.4	0.2	0.0	1.4	1.1
6	*0.54*	*0.55*	*51.4*	*0.60*	*0.61*	*52.6*	*0.65*	*0.71*	*56.6*	0.0	0.1	0.0	0.0	0.0	0.3	0.0	0.0	0.5	0.1
0	0.00	0.00	0.0	0.00	0.00	0.0	0.00	0.00	0.0	14.9	14.7	23.3	4.3	14.9	14.7	23.3	3.7	23.0	11.3
**Scenario 14**																			
1	*0.02*	*0.18*	*50.0*	*0.04*	*0.15*	*47.4*	***0.06***	***0.45***	***64.6***	**5.0**	**4.9**	**13.7**	**85.9**	**4.6**	**4.1**	**10.0**	**58.4**	**14.8**	**90.4**
2	**0.08**	**0.35**	**57.8**	0.24	0.25	45.4	*0.52*	*0.46*	*46.8*	4.1	5.9	6.4	6.2	4.4	7.1	9.1	21.9	5.6	3.8
3	*0.55*	*0.45*	*45.0*	***0.28***	***0.40***	***52.8***	*0.58*	*0.47*	*45.0*	3.1	2.7	1.9	0.6	3.2	2.4	2.1	2.4	1.9	0.4
4	*0.58*	*0.50*	*46.8*	*0.52*	*0.45*	*46.2*	*0.68*	*0.49*	*42.2*	2.0	0.8	0.4	0.0	1.9	0.5	0.1	0.0	0.6	0.2
5	*0.61*	*0.55*	*48.6*	*0.58*	*0.50*	*46.8*	*0.78*	*0.50*	*38.8*	0.8	0.2	0.0	0.0	0.7	0.1	0.0	0.0	0.2	0.0
6	*0.62*	*0.60*	*51.2*	*0.61*	*0.55*	*48.6*	*0.88*	*0.51*	*35.4*	0.0	0.0	0.0	0.0	0.0	0.0	0.0	0.0	0.0	0.0
0	0.00	0.00	0.0	0.00	0.00	0.0	0.00	0.00	0.0	14.9	14.5	22.4	7.3	14.9	14.3	21.4	17.3	23.2	5.2
**Scenario 15**																			
1	*0.02*	*0.18*	*50.0*	*0.04*	*0.15*	*47.4*	*0.06*	*0.01*	*38.2*	5.0	4.8	6.1	7.0	4.6	4.2	7.2	17.1	4.3	1.4
2	**0.08**	**0.35**	**57.8**	0.24	0.25	45.4	*0.52*	*0.05*	*22.2*	4.1	5.7	5.8	11.1	4.4	7.0	9.5	27.2	4.3	3.0
3	*0.55*	*0.45*	*45.0*	***0.28***	***0.40***	***52.8***	*0.58*	*0.25*	*31.8*	3.1	2.5	2.2	0.7	3.1	2.2	1.9	1.6	1.8	1.0
4	*0.58*	*0.50*	*46.8*	*0.52*	*0.45*	*46.2*	*0.68*	*0.35*	*33.8*	1.9	0.8	0.5	0.0	1.9	0.5	0.1	0.0	0.8	0.0
5	*0.61*	*0.55*	*48.6*	*0.58*	*0.50*	*46.8*	*0.78*	*0.45*	*35.8*	0.7	0.2	0.0	0.0	0.7	0.1	0.0	0.0	0.2	0.0
6	*0.62*	*0.60*	*51.2*	*0.61*	*0.55*	*48.6*	*0.88*	*0.50*	*34.8*	0.0	0.0	0.0	0.0	0.0	0.0	0.0	0.0	0.0	0.0
0	0.00	0.00	0.0	0.00	0.00	0.0	**0.00**	**0.00**	**0.0**	**14.8**	**14.1**	**14.7**	**81.2**	**14.8**	**14.0**	**18.7**	**54.1**	**11.4**	**94.6**
**Scenario 16**																			
1	*0.02*	*0.02*	*40.4*	*0.02*	*0.02*	*40.4*	*0.02*	*0.02*	*40.4*	3.0	0.1	0.1	0.0	3.0	0.0	0.0	0.0	3.0	0.1
2	*0.03*	*0.11*	*45.4*	*0.03*	*0.11*	*45.4*	*0.03*	*0.11*	*45.4*	3.0	0.5	0.5	0.5	3.0	0.2	0.2	0.6	3.1	1.4
3	0.05	0.22	51.2	0.05	0.22	51.2	0.05	0.22	51.2	3.0	1.8	3.8	5.3	3.0	1.2	1.7	6.6	3.5	9.0
4	**0.06**	**0.59**	**73.0**	**0.06**	**0.59**	**73.0**	**0.06**	**0.59**	**73.0**	**3.0**	**5.7**	**10.3**	**56.1**	**3.0**	**4.7**	**12.4**	**57.8**	**5.0**	**46.2**
5	0.25	0.59	65.4	0.25	0.59	65.4	0.25	0.59	65.4	3.0	5.5	7.8	35.8	3.0	6.0	8.7	32.2	5.0	33.6
6	*0.59*	*0.59*	*51.8*	*0.59*	*0.59*	*51.8*	*0.59*	*0.59*	*51.8*	0.0	1.1	1.0	0.4	0.0	2.7	0.4	0.5	4.2	7.5
0	0.00	0.00	0.0	0.00	0.00	0.0	0.00	0.00	0.0	14.9	14.8	23.5	1.9	14.9	14.7	23.5	2.3	23.8	2.2
**Scenario 17**																			
1	*0.01*	*0.07*	*43.8*	*0.01*	*0.07*	*43.8*	*0.01*	*0.07*	*43.8*	3.5	1.9	2.8	1.3	3.4	0.5	0.4	0.7	3.5	4.4
2	**0.05**	**0.56**	**71.6**	**0.05**	**0.56**	**71.6**	**0.05**	**0.56**	**71.6**	**3.8**	**5.2**	**8.6**	**54.9**	**3.9**	**6.6**	**13.1**	**58.4**	**6.7**	**48.4**
3	0.26	0.56	63.2	0.26	0.56	63.2	0.26	0.56	63.2	3.3	5.2	8.8	36.9	3.4	5.8	9.5	38.0	7.1	37.3
4	*0.48*	*0.56*	*54.4*	*0.48*	*0.56*	*54.4*	*0.48*	*0.56*	*54.4*	2.7	2.2	3.3	5.2	2.7	1.5	0.6	0.6	3.8	6.0
5	*0.55*	*0.58*	*52.8*	*0.55*	*0.58*	*52.8*	*0.55*	*0.58*	*52.8*	1.7	0.5	0.3	0.3	1.7	0.3	0.1	0.0	1.9	2.2
6	*0.59*	*0.61*	*53.0*	*0.59*	*0.61*	*53.0*	*0.59*	*0.61*	*53.0*	0.0	0.0	0.0	0.0	0.0	0.1	0.0	0.0	0.9	0.4
0	0.00	0.00	0.0	0.00	0.00	0.0	0.00	0.00	0.0	15.0	14.9	23.7	1.4	15.0	14.9	23.6	2.3	23.9	1.3
**Scenario 18**																			
1	*0.52*	*0.05*	*22.2*	*0.52*	*0.05*	*22.2*	*0.52*	*0.05*	*22.2*	4.1	0.6	0.1	0.3	4.0	0.4	0.1	0.0	4.5	1.5
2	*0.53*	*0.17*	*29.0*	*0.53*	*0.17*	*29.0*	*0.53*	*0.17*	*29.0*	1.6	0.4	0.1	0.0	1.6	0.3	0.1	0.0	1.9	1.5
3	*0.54*	*0.25*	*33.4*	*0.54*	*0.25*	*33.4*	*0.54*	*0.25*	*33.4*	0.8	0.2	0.1	0.0	0.8	0.2	0.1	0.0	0.8	0.7
4	*0.55*	*0.36*	*39.6*	*0.55*	*0.36*	*39.6*	*0.55*	*0.36*	*39.6*	0.3	0.1	0.0	0.0	0.3	0.0	0.0	0.1	0.3	0.3
5	*0.57*	*0.48*	*46.0*	*0.57*	*0.48*	*46.0*	*0.57*	*0.48*	*46.0*	0.1	0.0	0.0	0.0	0.1	0.0	0.0	0.0	0.1	0.0
6	*0.58*	*0.52*	*48.0*	*0.58*	*0.52*	*48.0*	*0.58*	*0.52*	*48.0*	0.0	0.0	0.0	0.0	0.0	0.0	0.0	0.0	0.0	0.0
0	**0.00**	**0.00**	**0.0**	**0.00**	**0.00**	**0.0**	**0.00**	**0.00**	**0.0**	**6.9**	**1.3**	**0.3**	**99.7**	**6.9**	**1.0**	**0.3**	**99.9**	**7.6**	**96.0**

*Note*: For each stage k =1, 2, 3, $\pi_{k}^{T}$, $\pi_{k}^{E}$, and $U_{k}$ are the true toxicity probability, efficacy probability, and mean utility. Sel = selection percentage at each dose. The true optimal dose in each scenario is given in boldface, and unacceptable doses are in italics. $n_{1}$ $n_{2}$, and $n_{3}$ denote the mean numbers of patients in stages 1, 2, and 3. “0” denotes no dose selected.

## 5 Sensitivity Analyses

We evaluated the performance of different patient subsample sizes across stages of the trial determined by tweaks. We kept the data‐generating scenarios 1–10 unchanged and only varied the stage‐wise allocation in order to compare the allocation to the primary 18/24 (*N* = 42) setting used in Section 4.1. Two alternative sample allocations for the one‐tweak scenarios were considered: 24/24 (*N* = 48) and 18/36 (*N* = 54). In practice, a given sample allocation between the stages is not planned initially, but rather is a consequence of when the tweak is made during the trial. The results are presented in the Supporting Information. When the dose–response curves change after the tweak, the 24/24 subsample sizes may underperform relative to 18/24, since 24/24 assigns a larger proportion of patients prior to the tweak, even with its larger total sample size. The 18/36 subsample sizes tend to yield the best performance among the three sample size distributions considered by allocating the greatest number of patients after the tweak, thereby improving the ability to learn about the updated manufacturing process. This highlights the fact that the design's performance is driven not only by the total sample size but also by how many patients are treated before and after the manufacturing tweak.

To assess robustness of BAR12 to prior specifications, we conducted a sensitivity analysis using four alternative prior specifications in which the priors of specific parameters are changed, denoted by Prior 1, 2, 3, and 4.

- Prior 1 (toxicity coefficient prior changed). Assume dose‐toxicity coefficient priors $\beta_{0}∼N\left(-4,{\sqrt{4}}^{2}\right)$ and $\beta_{1}∼N\left(1.1,{\sqrt{2}}^{2}\right)$.
- Prior 2 (efficacy coefficient prior changed). Assume dose‐efficacy parameter priors $\gamma_{0}∼N\left(-2,{\sqrt{5}}^{2}\right)$, $\gamma_{1}∼N\left(1,{\sqrt{1}}^{2}\right)$, $\gamma_{2}∼N\left(-5,{\sqrt{3}}^{2}\right)$, and $\gamma_{3}∼N\left(-1,{\sqrt{2}}^{2}\right)$.
- Prior 3 (weight parameter prior changed). Change the prior for the borrowing weight from Beta(1.0,1.0) to Beta(0.5,0.5).

**FIGURE 4:**
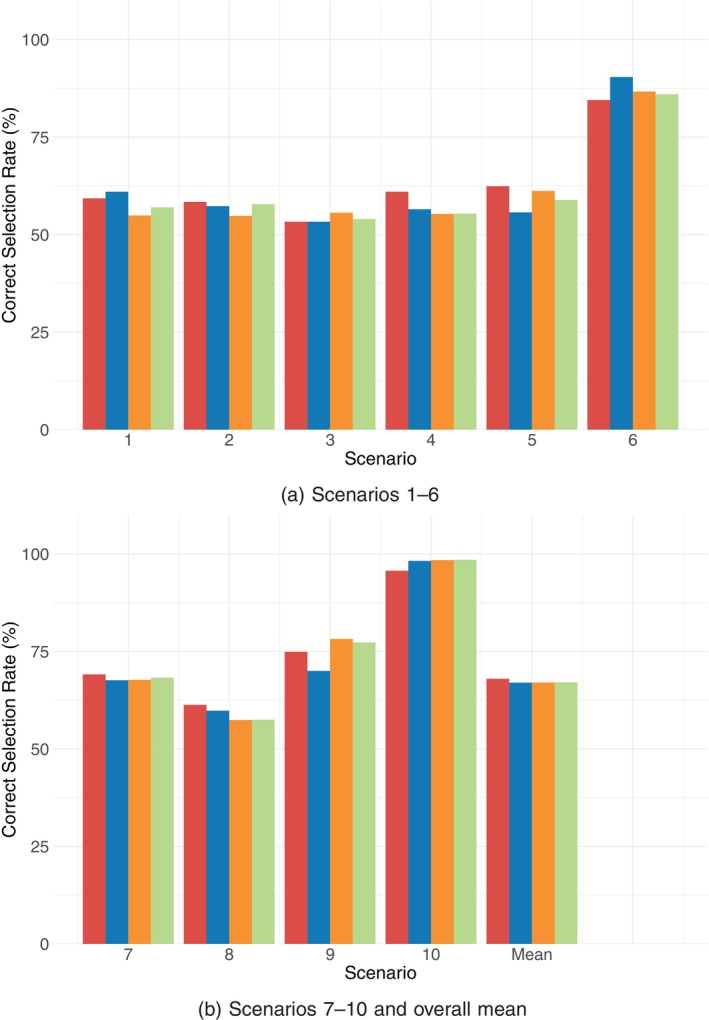
Sensitivity of the BAR12 design to the assumed prior, across ten scenarios. Each bar represents the correct selection rate under one of four prior specifications: Toxicity model (Prior 1, red), efficacy model (Prior 2, blue), weight parameter (Prior 3, orange), and original prior (green).

For each prior setting, all other priors were maintained as specified in Section 2.2. The prior means of the induced toxicity and efficacy probabilities are provided in the Supporting Information. The results are presented in Figure 4, where the results corresponding to priors 1, 2, 3, and the original prior are distinguished by the colors red, blue, orange, and green, respectively. The figure demonstrates that the proposed design is reasonably robust and performs well across these alternative prior settings.

We also conducted an additional sensitivity analysis to evaluate the robustness of the design to the frailty assumption by (1) retaining the normal frailty specification while varying the prior distribution for the frailty variance to assess sensitivity to latent heterogeneity, and (2) replacing the normal frailty with a Student‐t distribution to allow heavier tails. As shown in Section S2 of the Supporting Information, the design performance remains relatively stable under all frailty assumptions considered.

## 6 Discussion

We have proposed a new design, BAR12, that addresses problems arising from one or more interim manufacturing tweaks made to a newly engineered cellular therapy during a phase 1‐2 dose‐finding trial. The design exploits an assumed AR1 model to allow data obtained before a tweak to be used by the dose‐finding algorithm. This provides a practical alternative to either ignoring all data obtained before a tweak, or simply ignoring the fact that a tweak was made. The proposed AR1 modeling approach is broadly compatible with a variety of model‐based dose‐finding designs, in which the stage‐k parameter vector can be endowed with a mixture prior combining a spike at the posterior distribution from stage k−1 and a weakly informative slab component. However, because most model‐assisted designs, such as the widely used Bayesian optimal interval (BOIN) design [11, 24], do not specify parametric dose–response functions, applying the method in that setting is less straightforward and will require additional methodological development. We view this as an important open question for future research. Our simulations showed that, across a wide range of dose‐toxicity and dose‐efficacy scenarios, BAR12 is markedly superior to these simpler approaches. Given the increasing number of new cell therapies that must be tested, as well as other biotherapies, BAR12 provides a tool that may be used widely. It provides a scientifically valid alternative to the unrealistic requirement that a new trial must be started each time a manufacturing process is tweaked.

A practical advantage of BAR12 is that, because it is model‐based, the set of doses can differ across stages. For example, suppose that in manufacturing process 1 (stage 1) we are interested in six clinical doses $\left\{1.0,2.1,4.4,9.1,19.1,39.8\right\}\times 10^{6}$ cells, which we standardize to {0.0,0.2,0.4,0.6,0.8,1.0}. After a manufacturing tweak, suppose that the clinical team wishes to insert an additional intermediate dose of $27.5\times 10^{6}$ cells, so that the stage 2 dose set becomes {0.0,0.2,0.4,0.6,0.8,0.9,1.0}. Despite the change in the dose set, BAR12 operates without modification. A detailed specification of this scenario is provided in Section S3 of the Supporting Information. However, a more comprehensive evaluation of this broader application is warranted.

We have used prespecified, clinically interpretable scenarios, including deliberate misspecification, rather than a random‐scenario approach. To our knowledge, a validated bivariate outcome random‐scenario method for phase 1‐2 designs that jointly generates toxicity and efficacy probabilities is not yet available [25]. In addition, the specific problem we studied in this paper introduces further complications, as the manufacturing tweaks create another layer of dependence and heterogeneity that would need to be incorporated when generating random scenarios. Developing such a method within BAR12 is a natural direction for future work and may enable a more robust assessment of performance.

Accounting for patient heterogeneity in a phase 1‐2 trial may be very important. A very common setting is one where patients with several different indications are enrolled. While it may be realistic to assume that dose‐toxicity distributions are homogeneous for a given indication, the definitions of “response” and dose–response curves may be very different for different indications, or more generally for subgroups determined, for example, by prognosis or biomarkers. In general, accounting for heterogeneity requires a larger sample size to identify optimal subgroup‐specific doses reliably [17]. Extending BAR12 to accommodate tweaks when several patient subgroups are enrolled presents a challenge, since a given dose's desirability would depend on its toxicity rate, response rate, and manufacturing process for each subgroup. Key questions are whether BAR12 can be extended to accommodate such settings, and what sort of sample sizes may be required.

## Funding

This work was supported by National Institutes of Health, R01CA261978, R21LM014699, P01CA278716, and P30CA016672.

## Conflicts of Interest

The authors declare no conflicts of interest.

## Supporting information


**Data S1:** Supporting Information.

## Data Availability

Data sharing not applicable to this article as no datasets were generated or analyzed during the current study.
