# Supplementary material for: BAR12: Bayesian Autoregressive Phase 1‐2 Design for Cell Therapy Trials With Manufacturing Changes
Source: Stat Med. 2026 Apr 20;45:e70551. doi: 10.1002/sim.70551 (PMC13095505; doi:10.1002/sim.70551)
Supplement: Supplementary file 1 — Data S1: Supporting Information. [file SIM-45-0-s001.pdf]

Supplementary Materials for "A Bayesian Design for Early-Phase Cell Therapy Trials with Dynamic Manufacturing Changes"

Cheng-Han Yang[1], Peter F. Thall[1*], David Marin[2],
Sheferaw Y. Belay[1], and Ruitao Lin[1*]
[1]Department of Biostatistics,
[2]Department of Stem Cell Transplantation and Cellular Therapy,
The University of Texas MD Anderson Cancer Center, Texas 77030

S1 MCMC Convergence Evaluation

To evaluate whether Bayesian posterior estimation remains stable with seven model parameters per stage, we conducted an additional convergence assessment for the posterior distributions. At each decision time within each simulated trial, we fit the Bayesian model using four MCMC chains (10,000 iterations per chain, 5,000 warm-up iterations, with thinning by 2), yielding a total of 10,000 post–warm-up draws. We then computed the average Gelman–Rubin diagnostic, $\widehat{R}$, across all model parameters. For each scenario, we summarize the average $\widehat{R}$ at the end of Stage 1 and Stage 2. As shown in Table S5, all scenario- and stage-specific averages of $\widehat{R}$ fall between 1.0005 and 1.0013, which are well below commonly used thresholds (e.g., 1.05) and very close to the ideal value of 1. These results confirm that the MCMC chains converge reliably in both stages of the proposed design.

To further assess whether the posteriors are sufficiently concentrated for reliable inference, Tables S3–S4 summarize, for each key model parameter, the average ESS, average MCSE, average posterior standard deviation (SD), and average 95% credible interval (CI) width, along with the corresponding (lower, upper) bounds, at the end of Stage 1 and Stage 2 for each of the 10 scenarios. In Stage 2, we additionally report diagnostics for the two spike-and-slab weight parameters, ω^T and ω^E, which govern the degree of information borrowing from Stage 1 for the toxicity and efficacy submodels, respectively.

For the seven regression parameters $(\beta_0, \beta_1, \gamma_0, \gamma_1, \gamma_2, \gamma_3, \tau)$, the average ESS exceeds 5,000 in every scenario across both stages, indicating excellent mixing with negligible Monte Carlo error. The corresponding average MCSE values are uniformly small, confirming that posterior means are estimated with high precision. The posterior SD and CI width reflect the degree of posterior concentration for each parameter; narrower intervals at Stage 2 compared with Stage 1 indicate information gain as more patients are enrolled. As expected, posterior SDs generally show a greater reduction from Stage 1 to Stage 2 under Scenarios 7–10 (similar dose–outcome curves across stages) than under Scenarios 1–6 (dissimilar curves across stages), since the similarity across stages allows Stage 1 information to be effectively borrowed to improve Stage 2 estimation. The toxicity parameters β_0 and β_1 and the efficacy parameters γ_0 and γ_1 exhibit substantial narrowing of 95% CIs from Stage 1 to Stage 2, indicating strong posterior concentration for parameters governing the overall level and trend of each outcome. In contrast, the change-point parameters $(\gamma_2, \gamma_3, \tau)$ have relatively wider CIs at Stage 1, which narrow at Stage 2 as more data accumulate. This pattern is expected: with only 18 patients in Stage 1, the data are insufficient to separately identify

the three parameters that jointly govern the shape of the efficacy curve near its peak; however, by Stage 2, all parameters exhibit meaningful posterior concentration.

The weight parameters ω^T and ω^E exhibit excellent mixing in Stage 2, with average ESS values consistently exceeding 6,800 and average small MCSE across all 10 scenarios.

The patient-level variance parameter σ_ε^2 exhibits a notably lower average ESS (approximately 1,300–3,600, depending on the scenario and stage) and correspondingly higher MCSE than the regression parameters. This pattern is not unexpected given the small sample sizes in our trial: Stage 1 enrolls at most 18 patients, and the cumulative sample size by Stage 2 is at most 42. With such limited data, the variance component is inherently less well informed than the regression coefficients. As noted in the hierarchical modeling literature, variance (or scale) parameters are often more difficult to estimate in small samples, may be weakly identified, and can exhibit slower mixing than regression parameters (Gelman, 2006; Betancourt and Girolami, 2015). Nevertheless, the $\hat{R}$ value for σ_ε^2 remains close to 1 across all scenarios and stages, indicating satisfactory convergence. Importantly, dose-selection decisions depend on the overall shape of the fitted dose–response curve, obtained by integrating over patient-specific random effects, rather than on precise estimation of individual parameters. As demonstrated in Section 4, the design consistently selects doses within the optimal or near-optimal range.

Finally, trace plots for specific simulated trials can be used to visually assess chain mixing, and are reproducible using the `plot_mcmc_diagnostics()` function on our GitHub repository.

S2 Different Frailty Specifications

To evaluate the robustness of the proposed design to the frailty assumption, we examined the following additional frailty assumptions:

1. Retaining the normal distribution for the frailty parameter, but assigning a $\text{Unif}(0, 5)$ prior to the standard deviation σ_ϵ, replacing the original half-Cauchy prior;

2. Retaining the normal distribution for the frailty parameter, but assigning an Inverse-Gamma$(0.5, 0.5)$ prior to σ_ϵ^2;

3. Adopting a Student-t distribution with three degrees of freedom ($df = 3$) for the frailty parameter, while maintaining the Half-Cauchy$(0, 1)$ prior on σ_ϵ;

4. Adopting a Student-t distribution with seven degrees of freedom ($df = 7$) for the frailty parameter, again maintaining the Half-Cauchy$(0, 1)$ prior on σ_ϵ.

As shown in Figure S1a, the results obtained under the different frailty specifications are broadly consistent with those from the original model, indicating that the proposed design is robust to the frailty assumption.

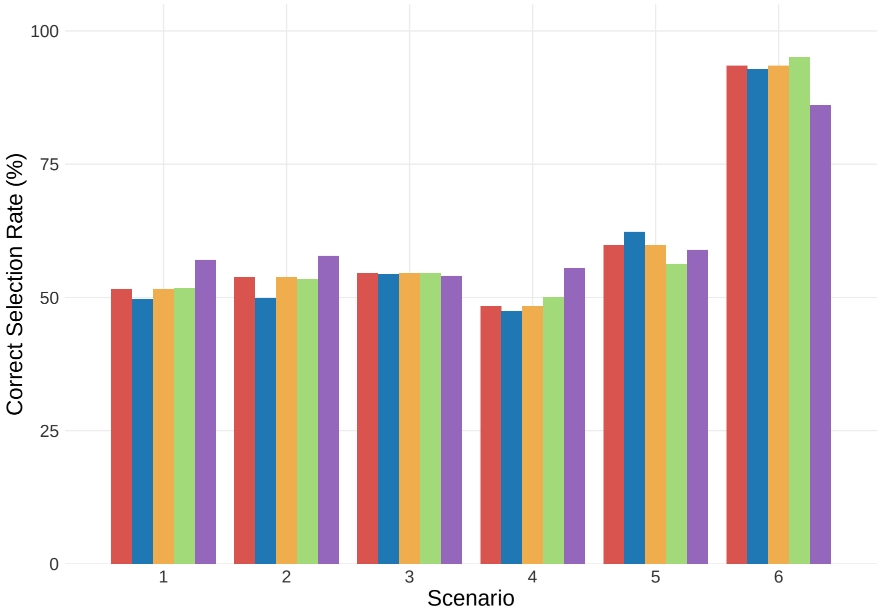

(a) Scenarios 1–6

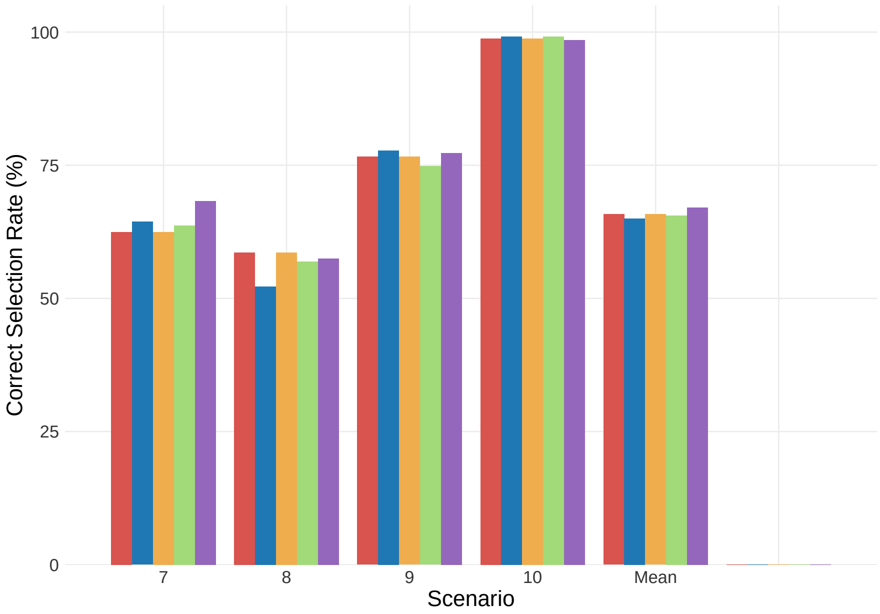

(b) Scenarios 7–10 and overall mean

Figure S1: Sensitivity of the BAR12 design to the frailty variance prior specification and frailty distribution specifications. Each bar represents the correct selection rate under a specific assumption: Unif(0, 5) prior for the frailty variance (Red), Inverse-Gamma(0.5, 0.5) prior for the frailty variance (Blue), Student's t-distributed frailty with $df = 3$ (Orange), Student's t-distributed frailty with $df = 7$ (Green), and the original frailty specification (Purple).

S3 Illustration of BAR12 with Different Dose Sets Across Stages

Since BAR12 is model-based, the set of available doses can differ across stages while inference is conducted on a common standardized dose scale. This section presents one simple scenario illustrating how the design accommodates the introduction of a new dose after a manufacturing change.

In stage 1, the design evaluates six standardized doses

$$x \in \{0.0, 0.2, 0.4, 0.6, 0.8, 1.0\},$$

with true toxicity and efficacy probabilities

$$\pi_1^T = (0.01,\ 0.02,\ 0.03,\ 0.03,\ 0.05,\ 0.38), \qquad \pi_1^E = (0.02,\ 0.03,\ 0.19,\ 0.25,\ 0.41,\ 0.41).$$

After a manufacturing tweak, stage 2 expands the dose set to

$$x \in \{0.0, 0.2, 0.4, 0.6, 0.8, 0.9, 1.0\},$$

by introducing a new intermediate dose at $x = 0.9$. The corresponding true toxicity and efficacy probabilities in stage 2 are

$$\pi_2^T = (0.02,\ 0.03,\ 0.04,\ 0.05,\ 0.06,\ 0.11,\ 0.35), \qquad \pi_2^E = (0.08,\ 0.12,\ 0.15,\ 0.25,\ 0.35,\ 0.65,\ 0.35).$$

The underlying dose–toxicity and dose–efficacy relationships for this scenario are shown in Figure S2. Based on Stage 1 alone, where $x = 0.9$ is not available, the dose–efficacy curve appears nearly flat between $x = 0.8$ and $x = 1.0$, suggesting a plateau. After introducing the new dose $x = 0.9$ in Stage 2, the unimodal shape of the true dose–efficacy curve becomes apparent, with its peak at $x = 0.9$.

We next consider a single illustrative implementation of BAR12 under this scenario. In this run, a total of 18 patients are treated in Stage 1 and 24 in Stage 2 (18/24). The implementation is summarized in Table S7. In Stage 1 ($k = 1$), the trial starts at the lowest standardized dose $x = 0.0$ and, subject to the toxicity and efficacy monitoring rules, enrolls patients sequentially at each higher dose in turn, escalating through the available doses up to $x = 1.0$. In Stage 2 ($k = 2$), after introducing $x = 0.9$, BAR12 restricts further enrollment to doses that are not excluded by the toxicity or futility rules and, at each decision point, allocates patients among these acceptable doses according to their posterior utilities. Over time, allocation concentrates on the new dose $x = 0.9$, which attains the largest posterior utility while remaining neither overly toxic nor futile. In this illustrative run, BAR12 therefore recommends $x = 0.9$ as the optimal dose at the end of the trial.

References

Betancourt, M. and M. Girolami (2015). Hamiltonian monte carlo for hierarchical models. *Current trends in Bayesian methodology with applications 79*(30), 2–4.

Gelman, A. (2006). Prior distributions for variance parameters in hierarchical models (comment on article by Browne and Draper). *Bayesian Analysis 1*(3), 515–534.

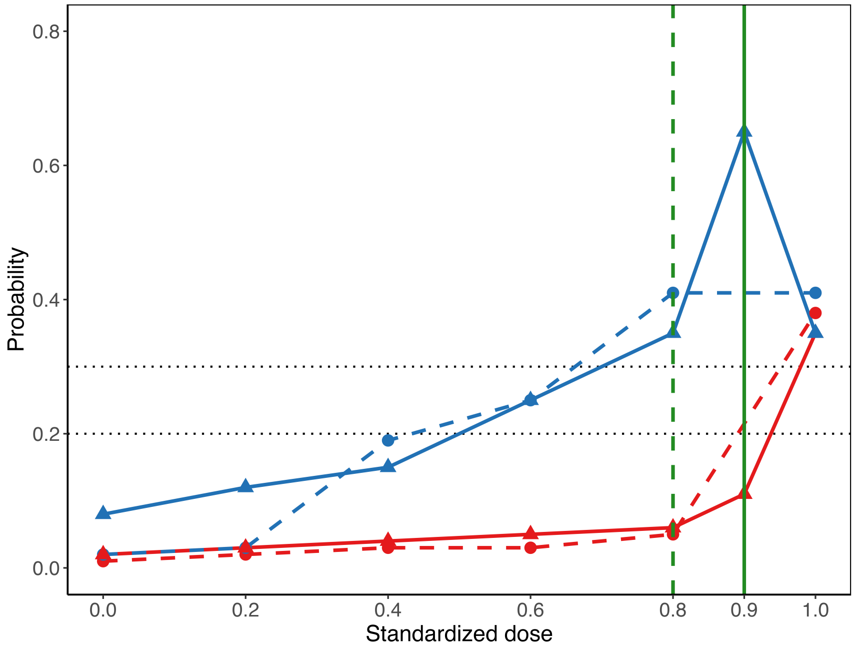

Figure S2: True dose–toxicity and dose–efficacy curves in the illustrative scenario with six standardized doses in stage 1 and seven standardized doses in stage 2, after adding $x = 0.9$. The red and blue curves represent the true probabilities of toxicity (π^T) and efficacy (π^E), respectively. Dashed lines with circular markers correspond to stage 1, while solid lines with triangular markers correspond to stage 2 after the tweak. Horizontal dotted lines identify the upper toxicity probability limit $\overline{\pi}^T = 0.3$ and the minimum clinically acceptable efficacy probability $\underline{\pi}^E = 0.2$, and the vertical green dashed and solid lines mark the true optimal doses for processes 1 and 2, respectively.

Table S1: Parameter sets for the illustrative curves in Figure 1.

**Profile Type**	**Curve Color**	γ_0	γ_1	γ_2	γ_3	τ
Monotone Increasing	Blue	-2	log(2)	0	0	0.5
	Red	-2	log(1)	6	0	0.5
Plateau	Blue	-2	log(10)	-10	0	0.2
	Red	-2	log(6)	-6	0	0.6
Unimodal	Blue	-2	log(10)	-12	1	0.2
	Red	-2	log(7)	-9	-1.5	0.8

Table S2: Prior means and variances of induced toxicity and efficacy probabilities under different prior specifications. The Original specification corresponds to the baseline prior configuration detailed in Section 2.2. Priors 1 – 4 correspond to the alternative prior settings presented in Section 5.

		Dose Level					
Specification	Type	1	2	3	4	5	6
**Prior Mean**							
Original	Toxicity	0.174	0.311	0.407	0.477	0.533	0.577
	Efficacy	0.380	0.512	0.575	0.605	0.614	0.609
Prior 1	Toxicity	0.096	0.218	0.314	0.388	0.448	0.497
	Efficacy	0.380	0.512	0.575	0.605	0.614	0.609
Prior 2	Toxicity	0.174	0.311	0.407	0.477	0.533	0.577
	Efficacy	0.236	0.324	0.393	0.438	0.461	0.468
Prior 3	Toxicity	0.216	0.339	0.425	0.490	0.541	0.582
	Efficacy	0.397	0.513	0.572	0.601	0.611	0.608
Prior 4	Toxicity	0.174	0.311	0.407	0.477	0.533	0.577
	Efficacy	0.380	0.512	0.575	0.605	0.614	0.609
**Prior Variance**							
Original	Toxicity	0.065	0.122	0.150	0.162	0.166	0.165
	Efficacy	0.124	0.144	0.150	0.153	0.159	0.167
Prior 1	Toxicity	0.035	0.097	0.137	0.159	0.170	0.176
	Efficacy	0.124	0.144	0.150	0.153	0.159	0.167
Prior 2	Toxicity	0.065	0.122	0.150	0.162	0.166	0.165
	Efficacy	0.087	0.115	0.136	0.150	0.160	0.168
Prior 3	Toxicity	0.095	0.141	0.163	0.172	0.174	0.173
	Efficacy	0.143	0.158	0.162	0.164	0.168	0.174
Prior 4	Toxicity	0.065	0.122	0.150	0.162	0.166	0.165
	Efficacy	0.124	0.144	0.150	0.153	0.159	0.167

Table S3: MCMC diagnostics and posterior uncertainty for each parameter under Scenarios 1–5: average effective sample size (ESS), average Monte Carlo standard error (MCSE), average posterior standard deviation (SD), and average 95% credible interval (CI) width with (lower, upper) bounds at the end of Stage 1 and Stage 2. All quantities are averaged over 1,000 replications, each based on 10,000 post–warm-up posterior draws.

	End of Stage 1				End of Stage 2			
Parameter	ESS	MCSE	SD	CI Width (Lower, Upper)	ESS	MCSE	SD	CI Width (Lower, Upper)
Scenario 1								
β_0	6,305	0.020	1.36	5.29 (−7.54, −2.26)	6,643	0.014	1.08	4.23 (−6.36, −2.13)
β_1	6,902	0.012	0.93	3.57 (−1.97, 1.60)	7,219	0.008	0.69	2.67 (−1.09, 1.58)
γ_0	6,537	0.017	1.28	5.00 (−5.99, −0.99)	7,770	0.014	1.20	4.73 (−4.54, 0.19)
γ_1	6,006	0.012	0.90	3.48 (−1.28, 2.21)	7,810	0.008	0.66	2.57 (−0.71, 1.86)
γ_2	8,092	0.024	1.74	6.83 (−8.25, −1.42)	8,542	0.016	1.40	5.52 (−7.63, −2.11)
γ_3	7,625	0.018	1.33	5.20 (−2.29, 2.91)	8,315	0.012	1.04	4.09 (−1.78, 2.31)
τ	6,847	0.005	0.38	1.39 (0.08, 1.47)	7,764	0.003	0.21	0.90 (0.37, 1.27)
σ^2_ε	1,305	0.155	4.18	11.90 (0.09, 11.99)	1,604	0.097	3.34	8.06 (0.05, 8.11)
ω^T	—	—	—	—	8,229	0.003	0.27	0.93 (0.03, 0.96)
ω^E	—	—	—	—	8,046	0.003	0.27	0.93 (0.03, 0.96)
Scenario 2								
β_0	6,243	0.019	1.35	5.26 (−7.49, −2.22)	6,658	0.013	1.04	4.04 (−5.60, −1.56)
β_1	6,940	0.012	0.93	3.58 (−1.97, 1.61)	7,963	0.008	0.68	2.63 (−0.83, 1.80)
γ_0	6,568	0.016	1.25	4.90 (−5.82, −0.92)	7,916	0.011	0.97	3.83 (−3.98, −0.15)
γ_1	6,201	0.012	0.91	3.53 (−1.36, 2.17)	8,415	0.008	0.69	2.66 (−0.88, 1.78)
γ_2	8,172	0.023	1.74	6.80 (−8.31, −1.51)	8,624	0.015	1.38	5.45 (−7.78, −2.33)
γ_3	7,734	0.017	1.33	5.20 (−2.48, 2.72)	8,563	0.011	1.03	4.05 (−2.15, 1.91)
τ	7,202	0.005	0.37	1.37 (0.09, 1.46)	8,349	0.002	0.21	0.88 (0.35, 1.23)
σ^2_ε	1,399	0.109	3.52	10.80 (0.11, 10.91)	1,618	0.097	3.47	8.55 (0.06, 8.60)
ω^T	—	—	—	—	8,183	0.003	0.26	0.93 (0.04, 0.97)
ω^E	—	—	—	—	8,141	0.003	0.26	0.92 (0.03, 0.96)
Scenario 3								
β_0	6,355	0.018	1.40	5.42 (−7.23, −1.81)	7,505	0.013	1.11	4.32 (−6.20, −1.88)
β_1	6,059	0.012	0.84	3.25 (−1.30, 1.94)	7,567	0.007	0.61	2.37 (−0.50, 1.87)
γ_0	6,777	0.017	1.32	5.13 (−5.98, −0.86)	8,117	0.013	1.14	4.47 (−4.33, 0.14)
γ_1	5,813	0.012	0.84	3.28 (−0.95, 2.33)	8,105	0.007	0.63	2.46 (−0.43, 2.02)
γ_2	8,107	0.021	1.74	6.83 (−8.28, −1.45)	8,681	0.015	1.40	5.51 (−7.69, −2.18)
γ_3	7,607	0.016	1.35	5.30 (−2.36, 2.93)	8,508	0.012	1.06	4.18 (−1.87, 2.31)
τ	7,124	0.005	0.37	1.37 (0.09, 1.47)	8,162	0.002	0.21	0.87 (0.40, 1.27)
σ^2_ε	1,345	0.114	4.93	10.47 (0.08, 10.54)	1,530	0.149	3.60	10.46 (0.06, 10.52)
ω^T	—	—	—	—	8,497	0.003	0.27	0.94 (0.03, 0.96)
ω^E	—	—	—	—	8,157	0.003	0.26	0.93 (0.03, 0.96)
Scenario 4								
β_0	6,603	0.019	1.44	5.59 (−7.13, −1.54)	7,404	0.013	1.04	4.09 (−5.73, −1.65)
β_1	5,792	0.011	0.76	2.97 (−0.76, 2.21)	7,798	0.007	0.57	2.23 (0.08, 2.32)
γ_0	6,818	0.017	1.26	4.93 (−5.52, −0.59)	8,017	0.011	0.93	3.64 (−4.15, −0.51)
γ_1	5,966	0.012	0.86	3.35 (−1.01, 2.34)	8,181	0.007	0.63	2.45 (−0.22, 2.24)
γ_2	8,161	0.021	1.74	6.81 (−8.32, −1.51)	8,507	0.016	1.41	5.53 (−7.72, −2.18)
γ_3	7,817	0.017	1.36	5.33 (−2.52, 2.82)	8,446	0.012	1.10	4.32 (−2.01, 2.31)
τ	7,338	0.005	0.37	1.37 (0.09, 1.46)	8,372	0.002	0.22	0.89 (0.37, 1.26)
σ^2_ε	1,373	0.237	7.02	12.15 (0.06, 12.22)	1,565	0.104	3.45	9.55 (0.08, 9.63)
ω^T	—	—	—	—	8,241	0.003	0.28	0.94 (0.03, 0.96)
ω^E	—	—	—	—	8,193	0.003	0.27	0.93 (0.03, 0.95)
Scenario 5								
β_0	6,603	0.019	1.44	5.59 (−7.13, −1.54)	6,522	0.013	1.05	4.10 (−5.87, −1.77)
β_1	5,792	0.011	0.76	2.97 (−0.76, 2.21)	8,030	0.006	0.56	2.18 (0.18, 2.36)
γ_0	6,818	0.017	1.26	4.93 (−5.52, −0.59)	8,205	0.009	0.77	3.03 (−2.62, 0.41)
γ_1	5,966	0.012	0.86	3.35 (−1.01, 2.34)	8,505	0.008	0.72	2.80 (−0.90, 1.90)
γ_2	8,161	0.021	1.74	6.81 (−8.32, −1.51)	8,460	0.016	1.40	5.51 (−7.78, −2.28)
γ_3	7,817	0.017	1.36	5.33 (−2.52, 2.82)	8,512	0.012	1.09	4.31 (−2.36, 1.94)
τ	7,338	0.005	0.37	1.37 (0.09, 1.46)	8,112	0.003	0.27	1.04 (0.23, 1.27)
σ^2_ε	1,459	0.237	7.02	12.15 (0.06, 12.22)	1,565	0.063	2.35	7.75 (0.08, 7.83)
ω^T	—	—	—	—	8,221	0.003	0.28	0.94 (0.03, 0.96)
ω^E	—	—	—	—	7,724	0.003	0.26	0.91 (0.06, 0.97)

Table S4: MCMC diagnostics and posterior uncertainty for each parameter under Scenarios 6–10: average effective sample size (ESS), average Monte Carlo standard error (MCSE), average posterior standard deviation (SD), and average 95% credible interval (CI) width with (lower, upper) bounds at the end of Stage 1 and Stage 2. All quantities are averaged over 1,000 replications, each based on 10,000 post–warm-up posterior draws.

	End of Stage 1				End of Stage 2			
Parameter	ESS	MCSE	SD	CI Width (Lower, Upper)	ESS	MCSE	SD	CI Width (Lower, Upper)
Scenario 6								
β_0	6,501	0.017	1.30	5.06 (−6.01, −0.95)	7,900	0.011	0.97	3.81 (−4.99, −1.18)
β_1	6,153	0.012	0.89	3.44 (−0.87, 2.58)	8,264	0.007	0.66	2.55 (−0.17, 2.38)
γ_0	6,559	0.016	1.21	4.71 (−5.37, −0.66)	7,320	0.013	1.03	4.06 (−5.87, −1.81)
γ_1	6,557	0.013	0.99	3.80 (−1.38, 2.42)	8,511	0.009	0.77	3.00 (−1.21, 1.79)
γ_2	8,391	0.020	1.73	6.78 (−8.36, −1.58)	8,620	0.016	1.43	5.64 (−7.81, −2.17)
γ_3	8,235	0.017	1.38	5.42 (−2.74, 2.69)	8,637	0.013	1.14	4.48 (−2.44, 2.05)
τ	7,888	0.005	0.39	1.38 (0.08, 1.46)	8,508	0.003	0.26	1.04 (0.23, 1.27)
σ^2_ε	1,850	0.329	9.02	14.14 (0.08, 14.22)	1,753	0.049	2.05	6.00 (0.04, 6.04)
ω^T	—	—	—	—	8,133	0.003	0.28	0.94 (0.03, 0.96)
ω^E	—	—	—	—	7,261	0.004	0.29	0.95 (0.02, 0.97)
Scenario 7								
β_0	6,248	0.019	1.35	5.26 (−7.49, −2.22)	6,049	0.014	1.02	4.01 (−6.58, −2.57)
β_1	6,944	0.012	0.93	3.58 (−1.97, 1.61)	7,974	0.008	0.71	2.75 (−1.40, 1.36)
γ_0	6,567	0.016	1.25	4.90 (−5.82, −0.92)	7,794	0.013	1.12	4.44 (−4.34, 0.10)
γ_1	6,205	0.012	0.91	3.53 (−1.36, 2.17)	8,007	0.008	0.68	2.63 (−0.87, 1.76)
γ_2	8,169	0.023	1.74	6.80 (−8.31, −1.51)	8,468	0.015	1.39	5.48 (−7.73, −2.25)
γ_3	7,738	0.017	1.33	5.20 (−2.48, 2.71)	8,330	0.011	1.03	4.04 (−2.03, 2.01)
τ	7,205	0.005	0.37	1.37 (0.09, 1.46)	7,923	0.002	0.21	0.90 (0.34, 1.24)
σ^2_ε	1,304	0.096	3.46	8.54 (0.06, 8.59)	1,619	0.084	2.96	9.44 (0.07, 9.51)
ω^T	—	—	—	—	8,177	0.003	0.27	0.93 (0.02, 0.96)
ω^E	—	—	—	—	8,085	0.003	0.27	0.93 (0.03, 0.96)
Scenario 8								
β_0	6,090	0.020	1.39	5.39 (−6.81, −1.41)	7,006	0.013	1.04	4.06 (−5.91, −1.85)
β_1	5,958	0.011	0.81	3.14 (−0.79, 2.34)	7,887	0.007	0.62	2.42 (−0.19, 2.23)
γ_0	7,056	0.014	1.07	4.21 (−3.95, 0.26)	8,120	0.009	0.80	3.17 (−3.11, 0.07)
γ_1	6,416	0.013	0.93	3.59 (−1.33, 2.26)	8,340	0.008	0.70	2.72 (−0.74, 1.98)
γ_2	8,344	0.022	1.73	6.77 (−8.34, −1.57)	8,482	0.016	1.40	5.51 (−7.73, −2.22)
γ_3	8,142	0.019	1.36	5.34 (−2.70, 2.64)	8,431	0.012	1.09	4.30 (−2.21, 2.08)
τ	7,541	0.005	0.39	1.37 (0.08, 1.46)	8,353	0.003	0.23	0.92 (0.32, 1.24)
σ^2_ε	1,393	0.281	8.51	16.20 (0.08, 16.29)	1,598	0.082	2.83	8.67 (0.07, 8.75)
ω^T	—	—	—	—	8,373	0.003	0.28	0.94 (0.02, 0.96)
ω^E	—	—	—	—	8,114	0.003	0.27	0.93 (0.03, 0.96)
Scenario 9								
β_0	5,316	0.018	1.26	4.90 (−6.10, −1.20)	5,902	0.013	0.94	3.66 (−5.29, −1.62)
β_1	5,705	0.012	0.89	3.45 (−0.44, 3.01)	7,872	0.008	0.68	2.66 (0.41, 3.07)
γ_0	7,368	0.010	0.79	3.12 (−2.32, 0.80)	8,361	0.006	0.54	2.12 (−1.69, 0.43)
γ_1	7,551	0.013	1.04	4.01 (−1.81, 2.20)	8,494	0.009	0.83	3.20 (−1.27, 1.94)
γ_2	8,633	0.020	1.72	6.75 (−8.40, −1.64)	8,594	0.016	1.41	5.54 (−7.79, −2.24)
γ_3	8,526	0.017	1.39	5.46 (−2.90, 2.56)	8,531	0.013	1.14	4.49 (−2.43, 2.06)
τ	7,967	0.005	0.41	1.40 (0.05, 1.45)	8,500	0.003	0.23	0.95 (0.27, 1.22)
σ^2_ε	1,456	0.529	14.29	17.93 (0.09, 18.02)	1,691	0.133	4.19	10.06 (0.09, 10.15)
ω^T	—	—	—	—	7,796	0.003	0.28	0.94 (0.02, 0.96)
ω^E	—	—	—	—	7,901	0.003	0.27	0.93 (0.02, 0.96)
Scenario 10								
β_0	7,247	0.015	1.17	4.63 (−2.93, 1.70)	7,547	0.009	0.79	3.14 (−2.32, 0.83)
β_1	7,483	0.018	1.28	4.96 (−1.55, 3.41)	7,995	0.015	0.91	3.54 (−0.93, 2.61)
γ_0	6,415	0.019	1.42	5.58 (−5.45, 0.13)	6,472	0.012	0.93	3.67 (−4.18, −0.51)
γ_1	6,957	0.017	1.26	4.88 (−1.57, 3.31)	8,083	0.010	0.86	3.36 (−0.80, 2.56)
γ_2	8,172	0.021	1.72	6.75 (−8.36, −1.61)	8,328	0.016	1.41	5.56 (−7.75, −2.19)
γ_3	8,264	0.017	1.41	5.52 (−2.85, 2.68)	8,191	0.018	1.14	4.48 (−2.27, 2.22)
τ	7,588	0.005	0.41	1.40 (0.05, 1.46)	8,176	0.003	0.23	0.96 (0.29, 1.25)
σ^2_ε	2,643	0.696	31.22	27.79 (0.07, 27.86)	3,562	0.176	6.66	13.27 (0.08, 13.35)
ω^T	—	—	—	—	6,813	0.004	0.29	0.95 (0.02, 0.97)
ω^E	—	—	—	—	7,706	0.004	0.28	0.94 (0.02, 0.96)

Table S5: Mean (SD) of $\widehat{R}$ across 1000 simulations for each scenario examined in Section 4.

Scenario	End of Stage 1	End of Stage 2
1	1.0012 (0.0102)	1.0006 (0.0031)
2	1.0009 (0.0116)	1.0005 (0.0158)
3	1.0008 (0.0082)	1.0006 (0.0017)
4	1.0011 (0.0056)	1.0006 (0.0068)
5	1.0011 (0.0141)	1.0006 (0.0026)
6	1.0009 (0.0141)	1.0005 (0.0048)
7	1.0009 (0.0083)	1.0006 (0.0036)
8	1.0008 (0.0082)	1.0005 (0.0014)
9	1.0007 (0.0093)	1.0007 (0.0039)
10	1.0009 (0.0049)	1.0013 (0.0065)

Table S6: Simulation results for the BAR12, Identical, and Separate designs under Scenarios $1-5$. The designs were evaluated under two total sample sizes and allocation schemes: (1) N=48 with 24 patients in stage 1 and 24 in stage 2 (denoted 24/24), and (2) N=54 with 18 patients in stage 1 and 36 in stage 2 (denoted 18/36). Sel = Selection percentage at each dose. The true optimal dose in each scenario is given in boldface, and unacceptable doses are given in italics. n_1 and n_2 denote the mean numbers of patients in stages 1 and 2. "0" denotes no dose selected.

	24/24								18/36							
Dose	BAR12			Identical			Separate		BAR12			Identical			Separate	
Level	n_1	n_2	Sel	n_1	n_2	Sel	n_2	Sel	n_1	n_2	Sel	n_1	n_2	Sel	n_2	Sel
**Scenario 1**																
1	3.0	0.0	0.0	3.0	0.0	0.0	3.0	0.0	3.0	0.1	0.0	3.0	0.0	0.0	3.0	0.0
2	3.1	0.2	0.2	3.1	0.1	0.1	3.1	1.9	3.0	0.3	0.4	3.0	0.2	0.3	3.1	0.8
3	3.1	0.7	1.8	3.1	0.4	1.6	3.2	5.7	3.0	1.4	1.4	3.0	0.7	1.4	3.3	1.6
4	3.3	4.0	7.4	3.3	1.3	4.8	3.6	11.5	3.0	6.7	8.0	3.0	2.0	5.2	4.2	9.3
**5**	**3.5**	**10.8**	**56.9**	**3.5**	**3.2**	**19.4**	**4.3**	**28.8**	**3.0**	**16.0**	**60.1**	**3.0**	**8.1**	**33.0**	**7.9**	**41.5**
6	7.8	7.8	31.4	7.8	18.4	70.6	6.6	49.9	3.0	10.7	27.8	3.0	23.7	54.5	14.0	44.4
0	23.8	23.5	2.3	23.8	23.4	3.5	23.8	2.2	17.9	35.3	2.3	17.9	34.7	5.6	35.6	2.4
**Scenario 2**																
1	3.0	0.2	0.3	3.0	0.0	0.0	3.1	1.0	3.0	0.4	0.3	3.0	0.0	0.0	3.1	0.1
2	3.1	1.4	5.6	3.1	0.0	0.1	3.8	10.6	3.0	2.4	3.5	3.0	0.1	0.2	4.0	5.0
3	3.3	4.9	24.4	3.3	1.0	3.6	4.5	23.7	3.0	9.3	19.3	3.0	1.8	4.9	6.1	26.1
**4**	**3.8**	**8.0**	**54.4**	**3.8**	**5.6**	**30.8**	**5.0**	**43.8**	**3.0**	**12.7**	**65.2**	**3.0**	**14.5**	**51.0**	**9.4**	**49.6**
5	4.1	6.3	10.4	4.1	11.6	56.1	3.8	8.4	3.0	7.6	8.4	3.0	13.1	34.1	6.9	5.4
6	6.6	2.3	0.1	6.6	4.6	3.1	2.7	0.9	3.0	2.6	0.0	3.0	4.1	0.4	3.9	0.0
0	23.8	23.1	4.8	23.8	22.9	6.3	22.9	11.6	18.0	35.0	3.3	18.0	33.6	9.4	33.3	13.8
**Scenario 3**																
1	3.0	0.1	0.0	3.0	0.0	0.0	3.0	0.1	3.0	0.2	0.0	3.0	0.0	0.0	3.0	0.0
2	3.0	0.5	0.3	3.0	0.0	0.0	3.1	0.9	3.0	0.8	0.2	3.0	0.1	0.1	3.1	0.3
3	3.2	3.3	6.4	3.2	0.6	2.0	3.4	5.7	3.0	6.2	6.2	3.0	0.9	1.8	3.8	4.0
**4**	**4.2**	**8.2**	**50.5**	**4.2**	**7.8**	**42.0**	**4.9**	**38.9**	**3.1**	**13.2**	**59.7**	**3.1**	**12.8**	**48.8**	**9.5**	**52.1**
5	4.5	8.1	34.4	4.5	11.3	48.1	4.7	33.5	3.0	10.7	30.1	3.0	15.1	41.0	9.5	36.2
6	5.7	3.0	4.0	5.7	3.0	1.6	4.5	16.6	2.8	4.0	1.1	2.8	5.0	0.9	6.1	2.3
0	23.8	23.1	4.4	23.8	22.7	6.3	23.5	4.3	17.9	35.1	2.7	17.9	33.9	7.4	35.0	5.1
**Scenario 4**																
1	3.0	1.3	0.9	3.0	0.0	0.0	3.3	1.8	3.0	2.3	0.9	3.0	0.0	0.0	3.3	1.3
2	3.4	6.2	26.4	3.4	0.5	1.8	6.3	29.8	3.1	10.9	21.2	3.1	1.5	4.7	9.4	26.5
**3**	**4.3**	**7.7**	**55.3**	**4.3**	**8.4**	**52.6**	**7.4**	**47.1**	**3.3**	**11.5**	**60.3**	**3.3**	**15.5**	**54.8**	**13.5**	**52.5**
4	6.2	4.5	5.3	6.2	8.8	10.9	3.8	9.4	3.4	5.2	3.5	3.4	7.0	2.4	4.8	3.7
5	3.8	1.4	0.3	3.8	0.9	0.7	1.8	2.1	2.9	1.9	0.0	2.9	1.7	0.4	2.0	0.2
6	2.4	0.2	0.0	2.4	0.1	0.0	0.8	0.6	1.9	0.6	0.0	1.9	0.6	0.0	0.8	0.0
0	23.1	21.4	11.8	23.1	18.7	34.0	23.4	9.2	17.6	32.4	14.1	17.6	26.3	37.7	33.9	15.8
**Scenario 5**																
1	3.1	2.4	2.5	3.1	0.0	0.0	3.7	5.5	3.1	4.7	0.6	3.1	0.0	0.0	3.9	3.7
**2**	**3.4**	**6.8**	**60.2**	**3.4**	**1.1**	**5.8**	**7.1**	**67.0**	**3.1**	**11.8**	**69.2**	**3.1**	**2.7**	**10.1**	**11.0**	**72.6**
3	4.4	7.3	26.4	4.4	8.7	55.4	7.2	23.3	3.3	10.2	20.9	3.3	15.7	55.5	13.3	20.3
4	6.1	4.2	2.1	6.1	8.8	9.7	3.4	1.4	3.4	4.4	1.6	3.4	7.5	3.1	4.6	0.1
5	3.8	1.2	0.0	3.8	0.8	0.3	1.7	0.2	2.9	1.6	0.0	2.9	1.4	0.1	1.9	0.0
6	2.5	0.1	0.0	2.5	0.1	0.0	0.6	0.2	2.0	0.5	0.0	2.0	0.5	0.0	0.7	0.0
0	23.2	21.9	8.8	23.2	19.6	28.8	23.7	2.4	17.7	33.3	7.7	17.7	27.9	31.2	35.4	3.3

Table S6 (continued): Simulation results for the BAR12, Identical, and Separate designs under Scenarios 6–10. The designs were evaluated under two total sample sizes and allocation schemes: (i) N=48 with 24 patients in stage 1 and 24 in stage 2 (denoted 24/24), and (ii) N=54 with 18 patients in stage 1 and 36 in stage 2 (denoted 18/36). Sel = Selection percentages at each dose. The true optimal dose in each scenario is given in boldface, and unacceptable doses are in italics. n_1 and n_2 denote the mean numbers of patients in stages 1 and 2. “0” denotes no dose selected.

	24/24								18/36							
Dose	BAR12			Identical			Separate		BAR12			Identical			Separate	
Level	n_1	n_2	Sel	n_1	n_2	Sel	n_2	Sel	n_1	n_2	Sel	n_1	n_2	Sel	n_2	Sel
**Scenario 6**																
1	4.1	2.3	0.2	4.1	0.2	0.0	3.4	0.6	3.9	2.6	0.1	3.9	0.4	0.0	3.4	0.0
2	6.9	4.2	2.1	6.9	5.5	9.7	3.5	1.0	4.8	4.5	1.0	4.8	6.4	1.8	3.6	0.2
3	5.1	4.0	6.3	5.1	7.2	24.5	2.8	0.7	3.5	4.4	3.2	3.5	8.7	12.8	2.8	0.0
4	3.0	1.8	1.9	3.0	0.7	1.5	2.4	0.4	2.6	2.1	2.2	2.6	1.1	0.9	2.4	0.0
5	1.4	0.5	0.6	1.4	0.0	0.0	1.1	0.3	1.3	0.8	0.1	1.3	0.1	0.0	1.1	0.0
6	0.5	0.1	0.0	0.5	0.0	0.0	0.4	0.0	0.5	0.2	0.0	0.5	0.0	0.0	0.4	0.0
**0**	**21.0**	**13.0**	**88.9**	**21.0**	**13.5**	**64.3**	**13.6**	**97.0**	**16.5**	**14.6**	**93.4**	**16.5**	**16.7**	**84.5**	**13.8**	**99.8**
**Scenario 7**																
1	3.0	0.1	0.0	3.0	0.0	0.0	3.0	0.0	3.0	0.1	0.0	3.0	0.0	0.0	3.0	0.0
2	3.1	0.2	0.2	3.1	0.0	0.0	3.0	0.3	3.0	0.2	0.1	3.0	0.1	0.0	3.0	0.0
3	3.3	1.3	2.3	3.3	0.8	2.7	3.1	2.8	3.0	1.5	0.8	3.0	1.2	2.3	3.2	0.8
4	3.7	6.0	17.0	3.7	3.0	11.5	3.5	9.8	3.0	9.3	9.6	3.0	4.4	11.6	4.0	10.4
**5**	**4.1**	**10.3**	**66.9**	**4.1**	**8.7**	**43.0**	**4.1**	**28.7**	**3.0**	**16.2**	**76.0**	**3.0**	**12.4**	**44.7**	**6.6**	**34.7**
6	6.7	5.4	9.4	6.7	10.6	38.5	7.0	54.5	3.0	8.1	11.1	3.0	16.6	37.0	15.4	50.0
0	23.8	23.2	4.2	23.8	23.1	4.3	23.7	3.9	18.0	35.3	2.4	18.0	34.7	4.4	35.2	4.1
**Scenario 8**																
1	3.6	1.5	2.8	3.6	0.7	1.8	3.3	2.7	3.4	2.0	1.0	3.4	1.0	1.9	3.4	1.7
2	5.8	6.1	16.4	5.8	6.0	25.4	4.4	19.7	4.1	9.9	15.6	4.1	9.1	25.0	5.5	17.7
**3**	**6.3**	**8.8**	**55.9**	**6.3**	**13.3**	**62.1**	**6.9**	**51.0**	**3.9**	**13.8**	**66.5**	**3.9**	**19.5**	**63.3**	**13.4**	**62.9**
4	4.1	5.5	20.0	4.1	2.6	6.8	4.7	17.6	3.1	7.3	13.6	3.1	4.0	5.7	7.5	12.2
5	2.6	1.3	1.8	2.6	0.5	0.2	2.9	5.2	2.2	1.7	0.6	2.2	0.9	0.2	3.9	2.1
6	1.4	0.1	0.0	1.4	0.1	0.0	1.5	1.6	1.2	0.3	0.0	1.2	0.2	0.0	1.8	0.4
0	23.7	23.3	3.1	23.7	23.2	3.7	23.8	2.2	17.9	35.0	2.7	17.9	34.8	3.9	35.5	3.0
**Scenario 9**																
**1**	**12.9**	**12.2**	**79.4**	**12.9**	**17.6**	**78.4**	**10.3**	**72.1**	**8.9**	**19.5**	**83.8**	**8.9**	**26.4**	**79.6**	**16.0**	**77.1**
2	5.4	8.8	14.6	5.4	4.4	15.5	7.5	19.3	4.1	12.5	12.6	4.1	6.6	14.3	11.9	16.1
3	2.7	1.8	2.4	2.7	0.9	2.5	3.3	4.5	2.4	2.3	1.2	2.4	1.4	2.3	4.3	3.6
4	1.5	0.3	0.6	1.5	0.2	0.3	1.6	2.0	1.4	0.4	0.0	1.4	0.4	0.3	2.0	0.9
5	0.7	0.1	0.0	0.7	0.1	0.0	0.8	0.2	0.7	0.1	0.0	0.7	0.1	0.0	0.9	0.0
6	0.3	0.0	0.0	0.3	0.0	0.0	0.3	0.0	0.3	0.0	0.0	0.3	0.0	0.0	0.3	0.0
0	23.7	23.3	3.0	23.7	23.3	3.3	23.8	1.9	17.8	34.9	2.4	17.8	34.9	3.5	35.5	2.3
**Scenario 10**																
1	5.1	0.5	0.5	5.1	0.4	0.4	5.1	4.2	4.7	1.0	0.5	4.7	0.8	0.1	5.4	1.6
2	2.1	0.3	0.3	2.1	0.3	0.6	2.0	2.4	1.9	0.5	0.2	1.9	0.5	0.5	2.2	1.6
3	1.0	0.1	0.0	1.0	0.1	0.0	0.9	1.6	0.9	0.2	0.0	0.9	0.2	0.0	1.1	0.2
4	0.4	0.0	0.0	0.4	0.0	0.0	0.3	0.2	0.4	0.1	0.0	0.4	0.1	0.0	0.4	0.2
5	0.1	0.0	0.0	0.1	0.0	0.0	0.1	0.0	0.1	0.0	0.0	0.1	0.0	0.0	0.1	0.0
6	0.0	0.0	0.0	0.0	0.0	0.0	0.0	0.1	0.0	0.0	0.0	0.0	0.0	0.0	0.1	0.0
**0**	**8.7**	**1.0**	**99.2**	**8.7**	**0.9**	**99.0**	**8.5**	**91.5**	**8.1**	**1.8**	**99.3**	**8.1**	**1.6**	**99.4**	**9.3**	**96.4**

Table S7: Illustrative BAR12 implementation with different numbers of standardized doses across stages. Stage 1 uses six doses $x_j \in \{0.0, 0.2, 0.4, 0.6, 0.8, 1.0\}$, whereas Stage $k = 2$ uses seven doses $x_j \in \{0.0, 0.2, 0.4, 0.6, 0.8, 0.9, 1.0\}$ after introducing a new dose at $x = 0.9$. For each stage k, decision, and standardized dose level x_j, n denotes the cumulative number of patients treated at x_j and $u(x_j, k, D_n)$ the posterior utility evaluated at x_j in stage k given the data set D_n based on n patients. In this illustrative run, a total of 18 patients are treated in stage 1 and 24 in stage 2 (18/24).

			Standardized dose level x_j						
Stage k	Decision		0.0	0.2	0.4	0.6	0.8	0.9	1.0
1	1	n	3	0	0	0	0	0	0
		$u(x_j, k, D_3)$	45.500	49.702	51.843	52.602	52.308	51.896	51.352
	2	n	3	3	0	0	0	0	0
		$u(x_j, k, D_6)$	42.646	43.532	44.035	43.934	43.324	42.857	42.302
	3	n	3	3	3	0	0	0	0
		$u(x_j, k, D_9)$	41.836	42.168	42.248	42.046	41.459	41.055	40.589
	4	n	3	3	3	3	0	0	0
		$u(x_j, k, D_{12})$	43.498	44.869	46.854	49.501	51.793	52.474	52.788
	5	n	3	3	3	3	3	0	0
		$u(x_j, k, D_{15})$	43.957	45.658	48.160	51.594	55.129	56.550	57.555
	6	n	3	3	3	3	3	0	3
		$u(x_j, k, D_{18})$	43.526	45.498	48.740	53.519	59.108	61.624	63.541
2	1	n	0	0	0	0	0	1	5
		$u(x_j, k, D_{24})$	49.004	50.931	53.031	54.881	55.579	55.026	53.754
	2	n	0	0	0	0	5	2	5
		$u(x_j, k, D_{30})$	49.494	51.652	54.089	56.321	57.182	56.531	55.127
	3	n	0	0	0	0	6	7	5
		$u(x_j, k, D_{36})$	52.818	55.798	59.276	62.759	64.901	64.795	63.654
	4	n	0	0	0	0	7	12	5
		$u(x_j, k, D_{42})$	52.668	55.846	59.638	63.631	66.596	66.967	66.211
